# The Extracellular Vesicles of the Helminth Pathogen, *Fasciola hepatica*: Biogenesis Pathways and Cargo Molecules Involved in Parasite Pathogenesis*[Fn FN2]

**DOI:** 10.1074/mcp.M115.053934

**Published:** 2015-10-20

**Authors:** Krystyna Cwiklinski, Eduardo de la Torre-Escudero, Maria Trelis, Dolores Bernal, Philippe J. Dufresne, Gerard P. Brennan, Sandra O'Neill, Jose Tort, Steve Paterson, Antonio Marcilla, John P. Dalton, Mark W. Robinson

**Affiliations:** From the ‡School of Biological Sciences, Queen's University Belfast, 97 Lisburn Road, Belfast, Northern Ireland;; §Área de Parasitología, Departamento de Biología Celular y Parasitología, Universitat de València, Burjassot, Valencia, Spain;; ¶Joint Research Unit on Endocrinology, Nutrition and Clinical Dietetics, Universitat de València-Health Research Institute La Fe, Valencia, Spain;; ‖Departmento de Bioquímica y Biología Molecular, Universitat de València, Burjassot, Valencia, Spain;; **Laboratoire de santé Publique du Québec, Sainte-Anne-de-Bellevue, Canada;; ‡‡School of Biotechnology, Dublin City University, Dublin 9, Republic of Ireland;; §§Departmento de Genética. Facultad de Medicina, UDELAR, Montevideo, Uruguay;; ¶¶Centre for Genomic Research, University of Liverpool, UK;; ‖‖Institute for Global Food Security, School of Biological Sciences, Queen's University Belfast, 97 Lisburn Road, Belfast, Northern Ireland, UK

## Abstract

Extracellular vesicles (EVs) released by parasites have important roles in establishing and maintaining infection. Analysis of the soluble and vesicular secretions of adult *Fasciola hepatica* has established a definitive characterization of the total secretome of this zoonotic parasite. *Fasciola* secretes at least two subpopulations of EVs that differ according to size, cargo molecules and site of release from the parasite. The larger EVs are released from the specialized cells that line the parasite gastrodermus and contain the zymogen of the 37 kDa cathepsin L peptidase that performs a digestive function. The smaller exosome-like vesicle population originate from multivesicular bodies within the tegumental syncytium and carry many previously described immunomodulatory molecules that could be delivered into host cells. By integrating our proteomics data with recently available transcriptomic data sets we have detailed the pathways involved with EV biogenesis in *F. hepatica* and propose that the small exosome biogenesis occurs via ESCRT-dependent MVB formation in the tegumental syncytium before being shed from the apical plasma membrane. Furthermore, we found that the molecular “machinery” required for EV biogenesis is constitutively expressed across the intramammalian development stages of the parasite. By contrast, the cargo molecules packaged within the EVs are developmentally regulated, most likely to facilitate the parasites migration through host tissue and to counteract host immune attack.

The trematode parasite, *Fasciola hepatica* is the causative agent of liver fluke disease (fasciolosis) in domestic animals. *F. hepatica* infects more than 300 million cattle and 250 million sheep worldwide resulting in losses of over $3 billion to global agriculture through lost productivity (1). Fasciolosis is also an important zoonotic disease with an estimated 2.6 million people infected worldwide; human infection is particularly highly prevalent in Egypt and the Andes of South America, while outbreaks regularly occur in Northern Iran (2).

Infection in animals and humans can last many years while adult parasites reside in the bile ducts. *F. hepatica* are obligate blood feeders and obtain blood by puncturing the bile duct wall. The regular regurgitation of parasite gut contents is thought to inject molecules into the bloodstream where they can exert an immunosuppressive activity on the host immune system (3). Other molecules are secreted from the gut, excretory pores and surface tegument into the bile where they may be carried to the exterior via the host intestine (4–6). We, and others, have previously used proteomic techniques to profile the secretome of adult *F. hepatica* (7–10) but these studies had a number of limitations: (1) they relied on very limited transcriptome data sets with considerable redundancy; (2) because genome sequence data was lacking it was not possible to determine precisely the identity and/or the number of the genes contributing to various secreted protein families; (3) the contribution of extracellular vesicles to fluke secretions was not investigated. However, our recent report of the *Fasciola* genome and associated transcriptome data sets (11) has now allowed us to address these limitations and perform a definitive characterization of the total secretome of adult *F. hepatica*. This is timely, given the report of exosome-like EVs released by adult *F. hepatica* (12).

Extracellular vesicles (EVs)^1^ are small membrane bound organelles that are shed by most cell types. Although once considered to be “cellular garbage cans” with the sole purpose of discarding unwanted cellular material (13), EVs are now recognized as important mediators of intercellular communication by transferring molecular signals, including proteins, lipids, mRNA, microRNA and other non-coding RNA species (14, 15). Variously described as exosomes or microvesicles depending on their cellular origin and mode of biogenesis, EVs perform a variety of roles in the maintenance of normal physiology such as blood coagulation, immune regulation and tissue repair (16–18), but also participate in pathological settings, notably in tumor progression (19). A number of recent studies have shown that parasite-derived EVs play an important role during infection. For instance, EVs released by the helminth parasites *Heligmosomoides polygyrus* and *Schistosoma japonicum* are capable of modulating the response of host innate immune cells (20, 21).

Here, we report the deep analysis of the soluble and vesicular components of the adult *F. hepatica* secretome. We found that *F. hepatica* secretes at least two subpopulations of EVs that differ according to size, cargo molecules and potential site of release from the parasite. Using a proteomic approach, integrated with newly available *F. hepatica* genome and transcriptome resources (11), we have defined the protein composition of these two classes of vesicle and non-vesicle associated proteins. The larger 15K EVs are released from the specialized cells that line the parasite gastrodermus and contain, as a marker, the 37 kDa inactive zymogen of the cathepsin L1 digestive peptidase. In contrast, our analysis of the smaller 120K exosome-like vesicle population suggest that they are shed from the tegument of adult fluke. Although the 120K EV biogenesis “machinery” is constitutively expressed across the intramammalian developmental stages of the parasite, the cargo molecules packaged within the EVs are highly regulated, most likely to facilitate the parasites migration through host tissue and to counteract host immune attack.

## EXPERIMENTAL PROCEDURES

### 

#### 

##### Experimental Design and Statistical Rationale

Western blots and fluorogenic assays were performed with at least two biological replicates and three technical triplicates. For the proteomics study, whole *Fasciola* EVs were initially analyzed by LC-MS/MS (two technical replicates). These identifications were subsequently mapped to the surface/membrane/lumen of the EVs following analysis of the EV fractions, which were run without replicates because of the paucity of parasite material. The aim of this experiment was to profile the molecules associated with fluke EVs and no quantitation was performed.

##### Isolation of Adult F. hepatica Extracellular Vesicles

Adult *F. hepatica* parasites were obtained from bovine livers from local abattoirs. To prepare secretions, adult flukes were thoroughly washed with PBS to void their gut contents and then maintained in RPMI 1640 culture medium containing 0.1% glucose, 100 U penicillin and 100 mg/ml streptomycin (Sigma), at 2 worms/ml for 5 h at 37 °C. EVs were purified according to the differential centrifugation protocol described by Marcilla *et al.* (12). Briefly, after the incubation period, the parasite culture media was collected and centrifuged at low speed (first at 300 × *g*/10 min, and then at 700 × *g*/30 min) to remove large debris. The resulting supernatant was centrifuged at 15,000 × *g* for 45 min at 4 °C to obtain large vesicles. Culture supernatants were then filtered using a 0.2 μm ultrafiltration membrane, and centrifuged at 120,000 × *g*/1 h at 4 °C to recover smaller vesicles that were subsequently washed with PBS.

##### Immunoblot Analysis of the EVs and Secretome

The protein content of adult fluke soluble secretions and EVs was determined using the BCA protein assay kit (Pierce, Loughborough, UK). Equal amounts (5 μg) of each sample were run on reducing NuPage Novex 4–12% Bis-Tris gels (Life Technologies), and transferred to nitrocellulose membranes (GE Healthcare, Paisley, UK) at 120 mA for 45 min. Following transfer, the membranes were incubated in blocking solution (TBST: 20 mm Tris-HCl, 150 mm NaCl, 1% Tween-20, pH 7.6) containing 5% skimmed milk for 2 h at room temperature (18–21 °C). Blots were probed with a 1:15,000 dilution of sheep antiserum raised against recombinant *F. hepatica* peroxiredoxin (FhPrx; 22) or recombinant cathepsin L1 (FhCL1; Dalton, unpublished); a 1:1000 dilution of rabbit antiserum raised against recombinant helminth defense molecule (FhHDM-1; 23) or a 1:5000 dilution of rabbit antiserum raised against recombinant *Schistosoma mansoni* leucine aminopeptidase which is reactive to *F. hepatica* leucine aminopeptidase (SmLAP; 24, Dalton unpublished) for 2 h at room temperature (18–21 °C). After washing in TBST (3 × 10 min), an appropriate alkaline phosphatase-conjugated IgG secondary antibody was applied to the membranes for 1 h at room temperature (18–21 °C) before detection using the BCIP/NBT substrate (Sigma).

##### Trypsin Shaving and Sequential Extraction of Purified F. hepatica EVs

Peptides from surface-accessible proteins were released by trypsin hydrolysis of EVs isolated from adult *F. hepatica* using our previously described protocol (25). Briefly, sequencing grade trypsin (Promega, Southampton, UK) was added to purified *F. hepatica* EVs at a final concentration of 50 μg/ml for 5 min at 37 °C. The treated EVs were then centrifuged at 100,000 × *g* for 1 h at 4 °C, and the released peptides recovered from the supernatant.

To investigate subvesicular protein localization, purified *F. hepatica* EVs were sequentially extracted to identify proteins associated with the EV membrane or those contained within the EV lumen. First, the EVs were resuspended in water; the resulting osmotic shock causes the EVs to burst and release the soluble contents held within the EV lumen. The EV membranes were collected by centrifugation at 100,000 × *g* for 1 h at 4 °C, while the soluble contents of the EV lumen were recovered in the supernatant. The membrane pellet was sequentially extracted with 0.1 m Na_2_CO_3_ (pH 11) on ice for 30 min to extract peripheral proteins and the sample centrifuged at 100,000 × *g* for 1 h at 4 °C. The final pellet was then solubilized in 1% Triton X-100/2% SDS for 15 min at 37 °C to produce integral membrane protein fractions (26).

##### Mass Spectrometry Analysis of EV and Secretome Fractions

Proteins in the various EV fractions were reduced with 2 mm DTT in 50 mm NH_4_HCO_3_ (60 °C/20 min) and alkylated with 5 mm iodoacetamide (room temperature (18–21 °C) in the dark/30 min). Samples were then incubated with 100 ng/μl sequencing grade trypsin (Promega) (37 °C/overnight). The digestions were stopped by the addition of trifluoroacetic acid (TFA) to a final concentration of 0.1% and dried in a vacuum centrifuge. The final mixtures were reconstituted with 10 μl of 0.1% TFA before analysis by LC-MS/MS. Five microliters of the resulting suspension were delivered to an analytical column (Eksigen C18-CL NanoLC Column, 3 μm; 75 μm × 15 cm) equilibrated in 5% acetonitrile/0.1% formic acid (FA). Elution was carried out with a linear gradient of 5–35% buffer B in buffer A for 30min (buffer A: 0.1% FA; buffer B: acetonitrile, 0.1% FA) at a flow rate of 300 nl/min. Peptides were analyzed in a nanoESI qQTOF mass spectrometer (5600 TripleTOF, ABSCIEX) operating in information-dependent acquisition mode, in which a 0.25-s TOF MS scan from 350–1250 *m*/*z*, was performed, followed by 0.05-s product ion scans from 100–1500 *m*/*z* on the 50 most intense 2–5 charged ions. Peak list files were generated by Protein Pilot v4.5 (Applied Biosystems) using default parameters and exported to Mascot v2.4.1 (Matrix Science) for database searching.

##### Database Searching

All MS/MS samples were analyzed using Mascot v2.4.1 (Matrix Science). Mascot was set up to search a database comprised of the gene models identified within the *F. hepatica* genome (version 1.0, 101,780 entries; 11) assuming trypsin digestion with 1 missed cleavage permitted. The *F. hepatica* gene model sequences can be accessed through WormBase ParaSite (http://parasite.wormbase.org/) under accession PRJEB6687 (genomic read data and gene model transcripts). Mascot was searched with a fragment ion mass tolerance of 0.1 Da and a parent ion tolerance of 0.1 Da. Iodoacetamide derivative of cysteine was specified in Mascot as a fixed modification. Glu->pyro-Glu of the N terminus, Gln->pyro-Glu of the N terminus, deamidation of Asn and Gln and oxidation of Met were specified in Mascot as variable modifications.

##### Criteria for Protein Identification

Scaffold (version Scaffold_4.3.0, Proteome Software Inc.) was used to validate MS/MS based peptide and protein identifications. Peptide identifications were accepted if they could be established at greater than 95.0% probability by the Peptide Prophet algorithm (27) with Scaffold delta-mass correction. Protein identifications were accepted if they could be established at greater than 95.0% probability and contained at least two identified peptides. Protein probabilities were assigned by the Protein Prophet algorithm (28). Proteins that contained similar peptides and could not be differentiated based on MS/MS analysis alone were grouped to satisfy the principles of parsimony.

##### Analysis of the F. hepatica Transcriptome and Gene Models

RNA sequencing and gene expression analysis was carried out for multiple lifecycle stages, including metacercariae, NEJ 1, 3 and 24 h postexcystment, juvenile 21-day-old and adult liver flukes, as described by Cwiklinski et al. (11). Briefly, Illumina TruSeq RNA libraries were prepared and sequenced, with the resulting RNAseq data being mapped to *F. hepatica* genome scaffolds and gene models. Differential expression analysis in edgeR was carried out for all genes to which at least five Illumina reads across all libraries could be mapped. Discovery of *F. hepatica* genes associated with EV machinery was carried out using BLAST analysis against the *F. hepatica* genome. Briefly, human proteins, inferred from the literature to have roles in exosome biogenesis, cargo sorting or uptake, were used as BLAST queries to identify homologs in closely-related trematode species (NCBI; trematoda). Both the human proteins and trematode homologs were used as BLAST queries to interrogate the *F. hepatica* genome (NCBI v2.2.29). Each match was manually validated and confirmed using InterPro (http://www.ebi.ac.uk/interpro/) which detects the presence of conserved protein domains. Expression of these identified genes across the *F. hepatica* lifecycle was investigated using the differential expression analysis of all the gene models identified within the genome. Hierarchical clustering using Gene Cluster 3.0 (29) was used to group differentially expressed genes by similarity of expression, graphically represented using heat-maps.

##### Analysis of Peptidase Activity in EVs

The soluble contents of *F. hepatica* EVs recovered following the 15,000 × *g* and 120,000 × *g* centrifugation of fluke culture media were isolated by osmotic shock. Briefly, the vesicle pellets were resuspended in hypotonic buffer (20 mm HEPES-KOH; pH 7.3) and placed on ice for 30 min with intermittent vortexing followed by sonication (5 × 20s at 20% power). The vesicle membranes were then removed by centrifugation at 20,000 × *g* for 1 h at 4 °C. Auto-activation of the native procathepsin L was carried out by incubating the soluble extract from the 15,000 × *g* vesicle pellet (50 μg total protein) at 37 °C in 100 mm citrate phosphate buffer, pH 4.5, containing 1 mm DTT. Aliquots (15 μl) were removed at time intervals and added to tubes containing 1 μl of 1 mm E-64 to stop the reaction. Proteolytic cleavage of the prosegment was visualized by immunoblotting as described above. Cathepsin L activity was also monitored in the presence of the fluorogenic peptide substrate Z-Leu-Arg-NHMec (20 μm) by measuring the release of fluorescence over time using a Polarstar Omega micro-plate reader (BMG Labtech) in 96-well fluorescent plates as previously described (30). Cathepsin B activity was measured by incubating the soluble vesicular extracts with Z-Arg-Arg-NHMec (10 μm) in 100 mm citrate-phosphate buffer (pH 6.5) containing 1 mm DTT and 0.01% Triton X-100. E-64 (10 μm) was used to inhibit cathepsin L and B activity. Leucine aminopeptidase activity was measured by incubating the vesicular extracts with the substrate H-Leu-NHMec (10 μm) in 100 mm Tris-HCl buffer (pH 7.5) containing 1 mm MnCl_2_ and 0.01% Triton X-100. Bestatin (50 μm), a broad-spectrum aminopeptidase inhibitor (31) was used to demonstrate inhibition of the reaction.

##### Transmission Electron Microscopy

Transmission electron microscopy (TEM) was performed on adult flukes as previously described (32). Briefly, adult flukes were fixed for 1 h in 2% double-distilled glutaraldehyde (DDG; Agar Scientific) in 0.1 m sodium cacodylate buffer (pH 7.2) containing 3% sucrose at 4 °C. After washing in buffer, specimens were dehydrated in ethanol and embedded in Agar 100 resin (Agar Scientific, Stansted, UK). Ultrathin sections (60–70 nm) were cut on a Reichert Ultracut E ultramicrotome and collected on bare 200-mesh nickel grids. For immunogold labeling, sections were etched with 10% hydrogen peroxide for 5 min then washed with 20 mm Tris-HCl buffer (pH 8.2) containing 0.1% bovine serum albumin and 2.5% Tween 20. Grids were incubated in normal goat serum (1:20 dilution in Tris-HCl buffer) for 30 min and then transferred to primary antibody diluted to 1:20,000 with 0.1% bovine serum albumin/Tris-HCl buffer for 12–18 h. Grids were washed in bovine serum albumin/Tris-HCl and then incubated with 10 nm gold-conjugated goat anti-sheep IgG (Bio Cell International) for 2 h at room temperature (18–21 °C). Grids were then lightly fixed with 2% DDG for 3 min, double stained with uranyl acetate and lead citrate and examined in a FEI (Philips) CM100 transmission electron microscope, operating at 100 keV. Controls included omission of primary antibody and incubation of grids with pre-immune serum followed by the secondary antiserum.

The exosome-like 120K vesicle pellet was analyzed by TEM as previously described (12). The 15K vesicle pellet was resuspended in PBS and aliquots placed on formvar-coated grids and examined in a FEI (Philips) CM100 transmission electron microscope, operating at 100 keV.

## RESULTS

### 

#### 

##### Profiling the Total Secretome of Adult F. hepatica

Taking advantage of our recently reported *F. hepatica* genome and transcriptome data sets for the major intramammalian life-cycle stages (newly excysted juveniles, NEJs; 21-day old immature liver stage juveniles, and adult flukes) (11), we were able to perform a definitive characterization of the secretions of adult *F. hepatica*. Sixty-nine proteins secreted by adult *F. hepatica* were identified in this analysis (supplemental Tables S1 and S2). In accordance with previous transcriptomic analyses (10, 11, 33) peptidases were highly represented (12 matches), including Pro-Xaa carboxypeptidases, cathepsin L1 and L2 clade members, dipeptidylpeptidase III, cathepsin A, legumain 2, metalloendopeptidases, and a cathepsin B. Inhibitors of serine peptidases, serpin, and cysteine peptidases, cystatin 1, were also identified. Another well represented functional group included molecules involved in defense against immune effector cells (10 matches), comprising helminth defense molecule (HDM), GST-mu and -sigma classes, fatty acid binding protein (FABP Fh 2), thioredoxin and thioredoxin-glutathione reductase. Other functional groups included membrane structure (12 matches), receptors/transporters (five matches), enzymes (seven matches), metabolism (six matches), and 14 hits corresponded to proteins with various/unknown functions. A large proportion of the identified proteins (30%) possessed a predicted N-terminal signal peptide, including the majority of the peptidases. Of the remaining proteins, 57% did not contain a predicted signal peptide, whereas 13% could not be predicted because of 5′ truncation of the transcripts.

##### Adult Fluke Culture Supernatants Contain Distinct Subpopulations of EVs

**Fig. 1.**
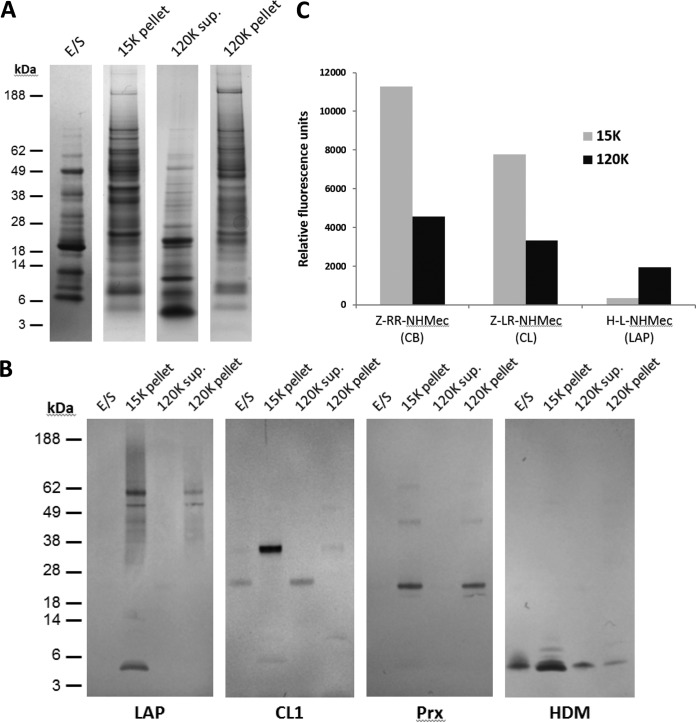
**Biochemical characterization of adult *F. hepatica* EV populations.** *A*, Typical 1-D profile of the adult *F. hepatica* total secretome (E/S), the 15 K pellet, the 120 K supernatant, and 120 K pellet. Proteins were separated using NuPage Novex 4–12% Bis-Tris gels (Life Technologies), and stained with colloidal Coomassie blue G250. *B*, Immunoblot analysis of the above fractions using antibodies against *F. hepatica* peroxiredoxin (Prx), cathepsin L1 (CL1), helminth defense molecule (HDM), and *S. mansoni* leucine aminopeptidase (LAP). C. Activity of soluble extracts from the 15 K and 120 K vesicle pellets against a panel of diagnostic fluorescent substrates that measure cathepsin B, cathepsin L and leucine aminopeptidase activity.

Following ultracentrifugation, a homogeneous group of exosome-like EVs (post-120,000 × *g* spin) 30–100 nm in diameter could be isolated from *F. hepatica* culture media as confirmed by transmission electron microscopy (supplemental Fig. S1*A*, and 12). However, in the present study we also recovered a pellet following the 15,000 × *g* centrifugation step that had been previously overlooked. Transmission electron microscopy also revealed that this pellet contained membrane-bound vesicles 50–200 nm in diameter (supplemental Fig. S1*B*–S1*E*). We performed a protein assay to determine the relative contributions of the 15 K pellet, the 120 K pellet, and the final post-120 K supernatant to the total protein secreted by adult flukes. We determined that adult *F. hepatica* secretes total protein at a rate of ∼17.3 μg/fluke/hr (100%) of which 0.6 μg/fluke/h (3.6%) is associated with the 120 K pellet, 1.5 μg/fluke/hr (8.6%) is associated with the 15 K pellet whereas the majority 15.2 μg/fluke/hr (87.8%) remains in solution following the 120 K spin. The complexity of each fraction, as determined by 1D gel electrophoresis, can be observed in Fig. 1*A*. Each sample is defined by a distinct protein profile indicating a compartmentalization of the total secretome that can be separated by centrifugation.

To investigate these fractions further, each fraction (5 μg total protein) was probed with specific antisera available in our laboratory against a range of secreted *Fasciola* secreted molecules (Fig. 1*B*). Immunoblotting showed that bands corresponding to helminth defense molecule (FhHDM; 6 kDa) were detected across all samples with the most intense HDM staining observed in the 15 K pellet. We found the *F. hepatica* leucine aminopeptidase (FhLAP; 55 kDa) and peroxiredoxin (FhPrx; 24 kDa) in both the 15 K and 120 K vesicle pellets but not in the total secretome or the 120 K supernatant. *F. hepatica* cathepsin L1 (FhCL1), which is synthesized as an inactive 37 kDa zymogen (9), was revealed as an intense reactive band in the 15 K vesicle pellet, but was barely visible by comparison in the 120 K pellet. Although the 37 kDa zymogen was not observed in the total secretome and final supernatant, a processed mature 24 kDa cathepsin L containing the catalytic domain was.

To confirm the above data, we used fluorogenic peptide substrates to determine the relative peptidase activities associated with the vesicle subpopulations (*i.e.* 15 K *versus* 120 K pellets). Although leucine aminopeptidase activity, measured using the substrate H-Leu-NHMec, was not detected in soluble extracts from either pellet, strong H-Leu-NHMec hydrolysis was observed when the membranes from both the 15 K and 120 K pellets were incubated with the substrate. The activity associated with membranes from the 120K pellet was fourfold higher than that associated with the 15 K pellet membranes in accordance with Acosta *et al.* (34). In all assays, this leucine aminopeptidase activity was inhibited by bestatin, a broad-spectrum aminopeptidase inhibitor (data not shown).

Equal amounts of total protein from each vesicle sample were incubated with diagnostic substrates for cathepsin L (Z-Leu-Arg-NHMec) and cathepsin B (Z-Arg-Arg-NHMec) activities. Comparison of the hydrolysis of each fluorogenic substrate within the linear range of the reactions (15 min) by each extract is shown in Fig. 1*C*. Strong cathepsin activity was detected in both 15 K and 120 K vesicle pellets. However, both cathepsin B- and L-specific activity in extracts of the 120 K pellet were on average 60% less than that observed in extracts of the 15 K pellet. In all assays, cathepsin B and L activity were completely inhibited by 10 μm E-64, a cysteine peptidase inhibitor (data not shown).

##### The 15 K EVs are Derived from Gastrodermal Cells and Contain Inactive Cathepsin L Zymogens that Can be Activated at low pH

**Fig. 2.**
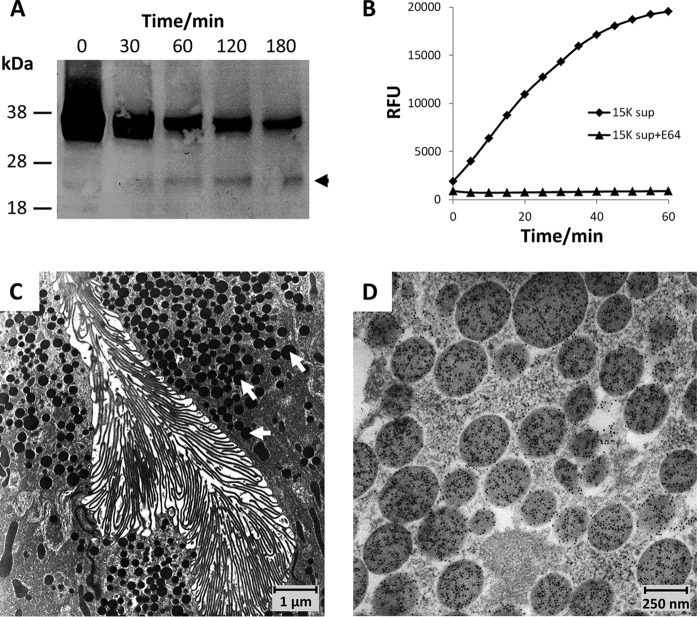
**Gastrodermal cell-derived EVs contain inactive cathepsin L zymogens.** *A*, Soluble extracts from the 15K vesicle pellet were incubated in 100 mm citrate phosphate buffer (pH 4.5) for 3 h at 37 °C. Aliquots of the reaction mixtures were removed after 0, 30, 60, 120, and 180 min and halted with 10 μm E-64 on ice. Samples were then transferred to nitrocellulose membranes and probed with an anti-*Fasciola* cathepsin L1 antibody. The inactive zymogen (37 kDa) showed a progressive decrease in intensity concurrent with the appearance of a band representing the 24 kDa mature enzyme (arrowhead). *B*, The auto-activation of FhCL1 shown in (*A*) was analyzed by following the hydrolysis of the fluorogenic dipeptide substrate Z-Leu-Arg-NHMec over 60 min at 37 °C. The autoactivation was inhibited in the presence of 10 μm E-64. *C*, Transmission electron micrograph (TEM) showing secretory vesicles (arrowed) within the specialized gastrodermal cells that line the gut of adult *F. hepatica. D*, TEM showing immunogold labeling for FhCL1 localized to the secretory vesicles within a gastrodermal cell within the gut of adult *F. hepatica*.

Our immunoblot analysis of the centrifugation fractions indicated that adult *F. hepatica* secretes a population of relatively large vesicles (15 K pellet) enriched for the inactive 37 kDa cathepsin L zymogen (Fig. 1*B*). To determine whether the native zymogen packaged within EVs could undergo pH-dependent autocatalytic activation similar to our purified recombinant cathepsin Ls (35–37), soluble extracts of the 15 K vesicle pellet were incubated at low pH (pH 4.5) for up to 3 h at 37 °C. Western blot analysis of the *in vitro* auto-activation process shows that the 37 kDa zymogen diminishes over time, concomitant with the appearance of a band corresponding to the processed 24 kDa mature enzyme (Fig. 2*A*; arrowed). The rate of formation of an active mature enzyme from the inactive zymogen at pH 4.5 was rapid indicating that autocatalytic activation of the native enzyme occurs readily in an acidic environment(Fig. 2B).

Transmission electron microscopy showed the presence of secretory vesicles densely packed within the specialized gastrodermal cells that line the fluke gut (Fig. 2*C*). Immunogold labeling using anti-cathepsin L antibodies revealed that these vesicles were intensely, and specifically, stained for *F. hepatica* cathepsin L1 (Fig. 2*D*). No labeling was observed in any other structures which indicates that the large vesicles of the 15 K pellet that are enriched for the 37 kDa cathepsin L1 zymogen originate from the gastrodermal cells of the fluke gut.

##### The Proteome of F. hepatica 120 K Exosome-like EVs

A proteomics analysis of the small exosome-like EVs (120 K vesicle pellet) was performed taking advantage of the newly available genome and transcriptome resources for *F. hepatica* (11), to gain a deeper coverage of the EV proteome than previously reported (12). Here, we performed a sequential treatment to identify differentially located proteins in the EVs. We first employed trypsin shaving (25) to release proteins from the surface of the EVs, before sequentially extracting them to identify proteins associated with the EV membranes as well as soluble proteins packaged within the EV lumen. A total of 180 different EV proteins were identified using this approach and their functional annotation is shown in Tables I and II.

**Table I:** Proteins associated with the membrane of EVs from adult F. hepatica. Figures represent number of unique peptides

Protein	Identifier	Extract		
		Trypsin	NaHCO_3_	TX100/SDS
*Proteases & inhibitors*				
Legumain 4/5	BN1106 s1861B000097	7	12	0
Cathepsin B	BN1106 s1772B000188	0	13	0
Cathepsin A	BN1106 s1241B000264	3	7	0
Cathepsin B6/8	BN1106 s793B000177	2	5	0
Cathepsin B fragment	BN1106 s8462B000006	0	4	0
Cathepsin B4/5/7	BN1106 s13444B000002	0	6	0
Cathepsin L1	BN1106 s8490B000026	0	5	0
Lysosomal Pro-X carboxypeptidase	BN1106 s1620B000120	0	4	0
M17 Leucine aminopeptidase 2	BN1106 s617B000566	0	6	0
20S proteasome subunit alpha 6	BN1106 s1579B000119	0	4	0
Proteasome beta 1 subunit	BN1106 s5855B000168	0	4	0
Proteasome subunit beta type-5	BN1106 s6770B000051	0	5	0
Proteasome subunit beta 2	BN1106 s2242B000188	0	5	0
Proteasome subunit alpha 7	BN1106 s7720B000057	0	3	0
Proteasome subunit alpha type	BN1106 s4981B000066	0	6	0
Proteasome subunit alpha type	BN1106 s3658B000104	0	4	0
20S proteasome subunit alpha 3	BN1106 s1639B000395	0	2	0
Lysosomal pro-Xaa carboxypeptidase	BN1106 s3518B000132	2	3	0
Proteasome subunit alpha 6	BN1106 s1259B000205	0	3	0
M17 Leucine aminopeptidase 1	BN1106 s7079B000034	0	2	0
20S proteasome subunit beta 2	BN1106 s2284B000154	0	4	0
Cathepsin L1	BN1106 s10332B000011	0	3	0
Multicystatin (cys1)	BN1106 s1612B000138	0	2	0
Proteasome subunit alpha type	BN1106 s9050B000016	0	2	0
*Pumps & transporters*				
ABCB1	BN1106 s274B000296	4	2	3
MDR p-glycoprotein 1	BN1106 s2471B000098	0	0	3
Glucose-transporter	BN1106 s584B000350	0	0	2
Phospholipid-translocating ATPase IIB	BN1106 s435B000242	0	0	3
ATP synthase subunit alpha	BN1106 s4332B000087	0	2	0
*Metabolism*				
Propionyl-CoA carboxylase alpha	BN1106 s1252B000359	17	3	0
Glutamate dehydrogenase 1 NAD(P)+	BN1106 s5767B000030	5	6	0
Aldolase	BN1106 s4469B000065	2	2	0
GAPDH	BN1106 s5174B000030	5	0	0
Glutamine synthetase	BN1106 s2400B000186	3	3	0
Propionyl-CoA carboxylase beta	BN1106 s1551B000468	0	3	0
Malic enzyme	BN1106 s233B000262	6	0	0
Hexokinase A	BN1106 s175B000200	0	0	2
Retinol dehydrogenase	BN1106 s2277B000049	0	0	2
Malate dehydrogenase	BN1106 s1459B000183	3	0	0
*Cytoskeleton*				
Actin 5C	BN1106 s2907B000133	11	4	0
Ezrin	BN1106 s1300B000145	2	0	2
Fimbrin	BN1106 s1403B000129	7	2	0
Alpha actinin	BN1106 s4069B000247	4	0	0
Gelsolin	BN1106 s2349B000188	3	2	0
Beta tubulin 1	BN1106 s55B000372	3	0	0
Rho1 GTPase	BN1106 s1908B000177	0	0	2
*Chaperones*				
Ubiquitin-60S ribosomal protein L40	BN1106 s6576B000103	0	2	2
Hsp70	BN1106 s309B000234	9	2	3
Chaperonin containing tcp1	BN1106 s1242B000159	2	0	0
14–3-3 protein	BN1106 s3904B000042	0	0	2
*Exosome biogenesis / vesicle trafficking*				
Annexin	BN1106 s819B000364	8	4	0
Tetraspanin-CD63 receptor	BN1106 s1657B000161	0	5	4
Acid sphingomyelinase	BN1106 s1285B000159	2	6	0
Annexin	BN1106 s945B000218	2	9	7
Myoferlin	BN1106 s3585B000136	0	0	11
Tetraspanin-1	BN1106 s915B000136	0	2	2
ALIX	BN1106 s2963B000136	0	0	6
IST1	BN1106 s3747B000112	2	0	7
Otoferlin	BN1106 s3261B000048	0	0	7
Vacuolar assembly/sorting protein D1D2-like protein	BN1106 s2316B000077	0	0	5
ALIX	BN1106 s1871B000313	0	0	4
Charged multivesicular body protein	BN1106 s2655B000264	0	0	4
Vacuolar protein sorting-associated protein 4B	BN1106 s1437B000141	0	0	4
Syntenin-1	BN1106 s4740B000062	0	0	3
Rab8	BN1106 s258B000276	0	0	2
CD63 antigen	BN1106 s4560B000072	0	2	0
Charged multivesicular body protein 5	BN1106 s6543B000070	0	0	5
Vacuolar protein sorting 26	BN1106 s1191B000313	0	0	2
Vacuolar protein sorting-associated protein VTA1	BN1106 s2858B000111	0	0	2
Annexin B2	BN1106 s500B000161	0	0	2
*Antioxidant/Defence*				
Peroxiredoxin	BN1106 s1614B000280	0	3	2
Secreted saposin-like protein SAP-3	BN1106 s10326B000017	0	4	0
Thioredoxin	BN1106 s4026B000080	0	3	2
GST sigma class	BN1106 s1081B000242	0	2	0
*Carrier proteins*				
Major vault protein	BN1106 s7273B000042	0	8	0
Ferritin	BN1106 s3950B000041	0	3	5
Niemann-Pick C2 protein	BN1106 s20469B000004	0	2	0
Ferritin	BN1106 s709B000627	0	5	0
Myoglobin	BN1106 s284B000287	0	3	2
*Enzymes*				
Phosphodiesterase-nucleotide pyrophosphatase	BN1106 s1985B000403	0	4	3
Carbonic anhydrase	BN1106 s3009B000044	0	3	3
Alpha mannosidase	BN1106 s666B000200	0	3	0
Ras-related protein Ral-A	BN1106 s637B000246	0	0	2
Alpha-galactosidase	BN1106 s1241B000260	0	2	0
*Uncharacterized / other*				
*F. hepatica* tegumental antigen-1	AF153056	6	0	3
DM9 domain-containing protein	BN1106 s5689B000026	2	4	4
Calmodulin-like protein	BN1106 s678B000118	0	2	0
Uncharacterized	BN1106 s3001B000132	0	5	5
Uncharacterized	BN1106 s8038B000016	0	4	7
Uncharacterized	BN1106 s4767B000028	0	2	3
Uncharacterized	BN1106 s6821B000024	0	2	4
Uncharacterized	BN1106 s440B000226	0	0	4
Uncharacterized	BN1106 s1110B000106	0	2	2
Uncharacterized	BN1106 s114B000615	0	2	2
Uncharacterized	BN1106 s4767B000027	0	0	2
Uncharacterized	BN1106 s4413B000122	0	4	0
Uncharacterized	BN1106 s551B000321	0	0	3
Uncharacterized	BN1106 s263B000609	0	4	0
Uncharacterized	BN1106 s3381B000140	0	2	0
Uncharacterized	BN1106 s739B000132	0	0	2

**Table II:** Top 50 proteins identified within the lumen of EVs released by adult F. hepatica

Protein ID	Identifier	Unique Peptides
*Proteases*		
Legumain 4/5	BN1106 s1861B000097	14
Cathepsin A	BN1106 s1241B000264	11
Cathepsin B	BN1106 s1772B000188	8
M17 Leucine aminopeptidase 2	BN1106 s617B000566	8
Cathepsin B6/8	BN1106 s793B000177	7
Cathepsin L1	BN1106 s8490B000026	7
20S proteasome subunit alpha 6	BN1106 s1579B000119	7
Cathepsin B4/5/7	BN1106 s13444B000002	6
Lysosomal Pro-X carboxypeptidase	BN1106 s1620B000120	6
Proteasome subunit beta type-5	BN1106 s6770B000051	6
20S proteasome subunit alpha 3	BN1106 s1639B000395	6
M17 Leucine aminopeptidase 1	BN1106 s7079B000034	5
*Membrane structure*		
Annexin	BN1106 s819B000364	17
Myoferlin	BN1106 s3585B000136	14
Tetraspanin-CD63 receptor	BN1106 s1657B000161	8
Annexin	BN1106 s945B000218	8
Tetraspanin-1	BN1106 s915B000136	8
Otoferlin	BN1106 s3261B000048	8
T-cell immunomodulatory protein	BN1106 s92B000560	7
Annexin	BN1106 s3266B000046	6
*EV biogenesis*		
ALIX	BN1106 s2963B000136	9
Acid sphingomyelinase	BN1106 s1285B000159	8
IST1	BN1106 s3747B000112	7
Vacuolar protein sorting-associated protein 4B	BN1106 s1437B000141	7
ALIX	BN1106 s1871B000313	6
Rab8	BN1106 s258B000276	6
Rab11b	BN1106 s844B000259	6
*Channels and transporters*		
ABCB1	BN1106 s274B000296	30
Na^+^/K^+^ transporting ATPase subunit alpha	BN1106 s521B000167	12
Niemann-Pick disease type C1 protein	BN1106 s1498B000257	10
MDR p-glycoprotein 1	BN1106 s2471B000098	8
vATPase subunit I	BN1106 s18772B000008	8
Inositol transporter	BN1106 s3611B000052	7
*Enzymes*		
Phosphodiesterase-nucleotide pyrophosphatase	BN1106 s1985B000403	9
Aldolase	BN1106 s4469B000065	9
Peroxiredoxin	BN1106 s1614B000280	5
Enolase	BN1106 s3227B000227	8
Glutamate dehydrogenase 1 NAD(P)+	BN1106 s5767B000030	7
Beta-1,4-galactosyltransferase 2	BN1106 s741B000214	6
*Uncharacterized/other*		
Major vault protein	BN1106 s7273B000042	15
Uncharacterized	BN1106 s8038B000016	11
Actin 5C	BN1106 s2907B000133	10
Uncharacterized	BN1106 s3001B000132	9
DM9 domain-containing protein	BN1106 s5689B000026	7
Uncharacterized	BN1106 s216B000184	7
Uncharacterized	BN1106 s6821B000024	6
Uncharacterized	BN1106 s440B000226	6
Ferritin	BN1106 s3950B000041	6
Ezrin	BN1106 s1300B000145	6
14–3-3 protein	BN1106 s3904B000042	5

A number of well-known exosomal markers, including Hsp70, ALIX and members of the tetraspanin family, were identified during the proteomics analysis. This supports the classification of the vesicles derived from the 120K pellet as “exosome-like.” Peptidases were highly represented in the EV proteome. Peptides corresponding to legumain 4/5, cathepsin A, cathepsin B6/8, and a Pro-Xaa carboxypeptidase were released following trypsin shaving of the EV surface. Other proteases, such as leucine aminopeptidase, various subunits of the *F. hepatica* proteasome and several cathepsin Bs were either found associated with the EV membrane (present in carbonate washes), or within the EV lumen (present in the soluble extract).

Several membrane channels, transporters and other molecules associated with membrane structure were identified. These included the ATPase pumps ABCB1/p-glycoprotein, Na^+^/K^+^ ATPase, and a phospholipid-transporting ATPase (flippase). Membrane transporters such as anoctamin (a calcium-activated chloride channel), Niemann-Pick C1, and C2 proteins (cholesterol transporters), an amino acid permease, an inositol transporter and a glucose transporter were also found. Other proteins with potential roles in membrane structure included various annexins and the *F. hepatica* T1 tegumental antigen (38). In addition to components of the cytoskeleton, peptides corresponding to several metabolic enzymes were released following trypsin shaving of the EV surface. These included propionyl-CoA carboxylase, glutamate dehydrogenase, glutamine synthase, malic enzyme, and malate dehydrogenase and GAPDH whereas other enzymes were found associated with the EV membrane or within the EV lumen. A small number of host-derived proteins were also identified including immunoglobulins, casein, and acylamino-acid-releasing enzyme (supplemental Table S3).

**Fig. 3.**
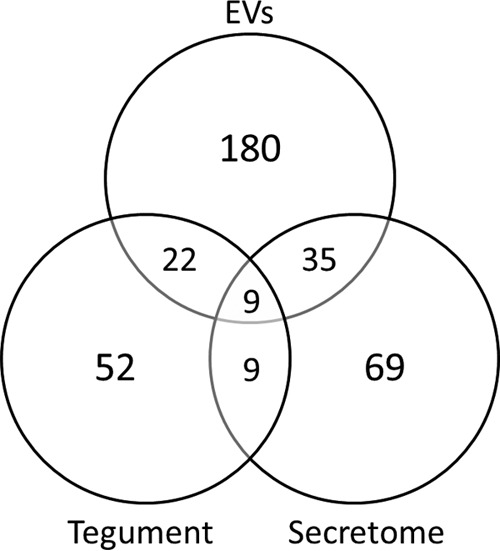
**Expression of proteins in the tegument, total secretome and EVs of adult *F. hepatica***. Venn diagram comparing the proteins identified in the total secretome and extracellular vesicles (EVs) released by adult *F. hepatica* (this study), and those found in tegumental fractions (tegument) by Wilson *et al.* (6).

Comparison of our data sets for adult fluke total secretome and the exosome-like EVs with the tegumental proteome reported by Wilson *et al.* (6) revealed that there is an overlap in protein expression between these samples (Fig. 3). Of the 180 proteins identified in the adult fluke EVs, 35 and 22 proteins were shared with the total secretome and tegument, respectively. A total of nine proteins were shared by the total secretome and tegument, whereas only nine proteins were found in all three samples. supplemental Fig. 2 shows the composition of the total secretome and EV proteome dats sets grouped according to function.

##### Transcriptome Profiling Reveals that Fasciola EV Biogenesis Pathways are Constitutively Expressed While Cargo Molecules are Developmentally Regulated

**Fig. 4.**
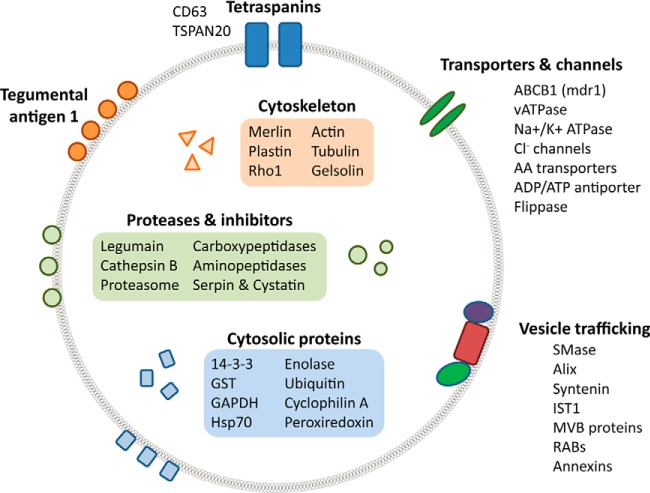
**Summary of the proteome of adult *F. hepatica* exosome-like EVs.**

A significant number of the proteins identified have roles in the endosomal pathway, or in EV biogenesis, and provide insight into the mechanism used by *F. hepatica* to generate EVs. These include various components of the ESCRT-dependent exosome biogenesis pathway (*e.g.* a number of charged multivesicular body proteins, the AAA-ATPase Vps4, IST1, syntenin and ALIX), and the ceramide-dependent pathway (*e.g.* sphingomyelinase). Several members of the Rab GTPase family, such as Rab8a, Rab11b, and Rab27 that have roles in EV biogenesis and release were also found. Also identified were SNAREs (synaptotagmin and VAMP7) as well as myoferlin and otoferlin that mediate vesicle trafficking and fusion. A cartoon summarizing the proteome of the exosome-like EVs from adult fluke is shown in Fig. 4 and the full mass spectrometry report is shown in supplemental Table S3.

To gain insight into the putative mechanism(s) of EV biogenesis in *F. hepatica* we conducted an extensive search of the literature for proteins implicated in this process via functional studies, largely in mammalian cells. A total of 102 proteins were identified and categorized according to their role in vesicle formation including: components of the endosomal sorting complexes required for transport (ESCRT) pathway, components of the ceramide pathway, roles in EV release (Rabs and SNAREs), RNA/protein/lipid sorting and membrane fusion/repair (supplemental Table 4). These proteins were used as queries to interrogate the newly available *F. hepatica* genome (11). All components of the ESCRT machinery, except for the charged multivesicular body protein 6 (CHMP6) from the ESCRT-III complex, were present in the *F. hepatica* genome. The ESCRT pathway was also well represented at the proteome level with up to ten putative members identified during the MS analysis of adult fluke exosome-like EVs; most of them were components of the ESCRT-III and Vps4 complexes that have roles in multivesicular body (MVB) formation and the final membrane budding/abscission steps during the latter stages of EV biogenesis (39). BLAST results also showed that many components of ESCRT-independent pathways for exosome release are present in the fluke genome but less widely represented at the proteome level. The most significant matches of the MS data include acid sphingomyelinase and tetraspanin CD63, that are both involved in exosome biogenesis in mammalian cells.

**Fig. 5.**
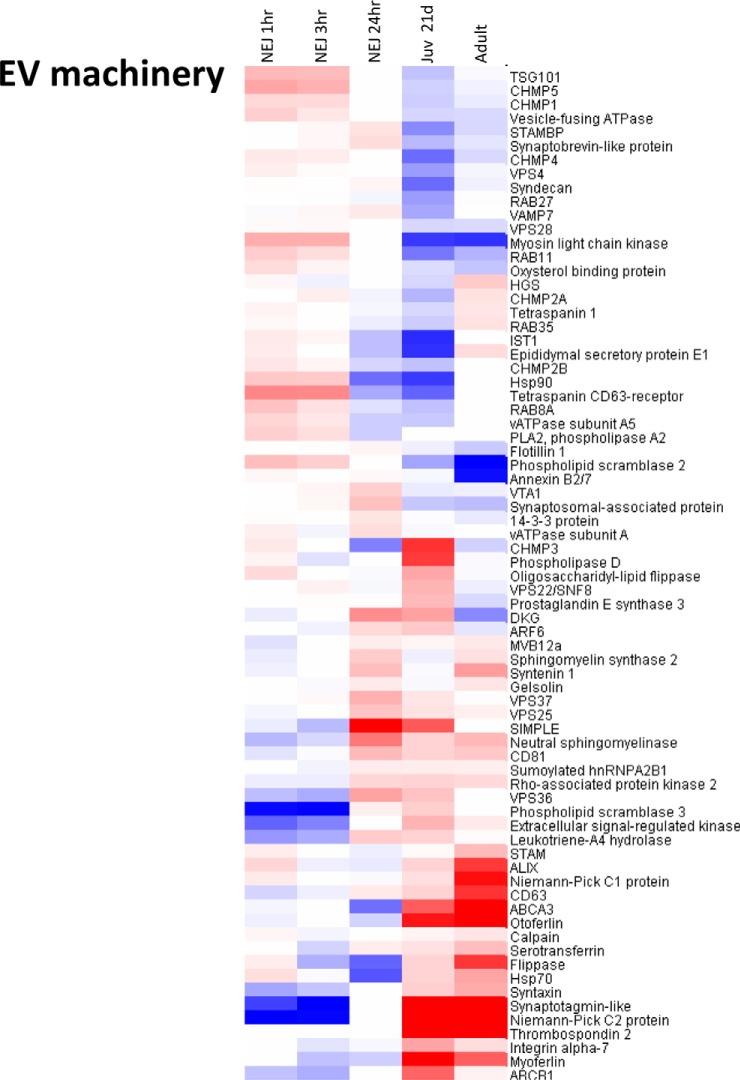
**Expression of transcripts encoding putative EV biogenesis pathway members across the intramammalian lifecycle stages of *F. hepatica***. Heatmaps are colored on a log_2_ scale with up-regulation in red and down-regulation in blue relative to metacercariae, depicting log fold change between developmental stages, grouped by hierarchical clustering. The *F. hepatica* life-cycle stages analyzed are: newly excysted juveniles (NEJ; 1-, 3- and 24-h postexcystment), 21-day old immature liver-stage flukes (Juv_21d), and adult flukes from the bile duct.

**Fig. 6.**
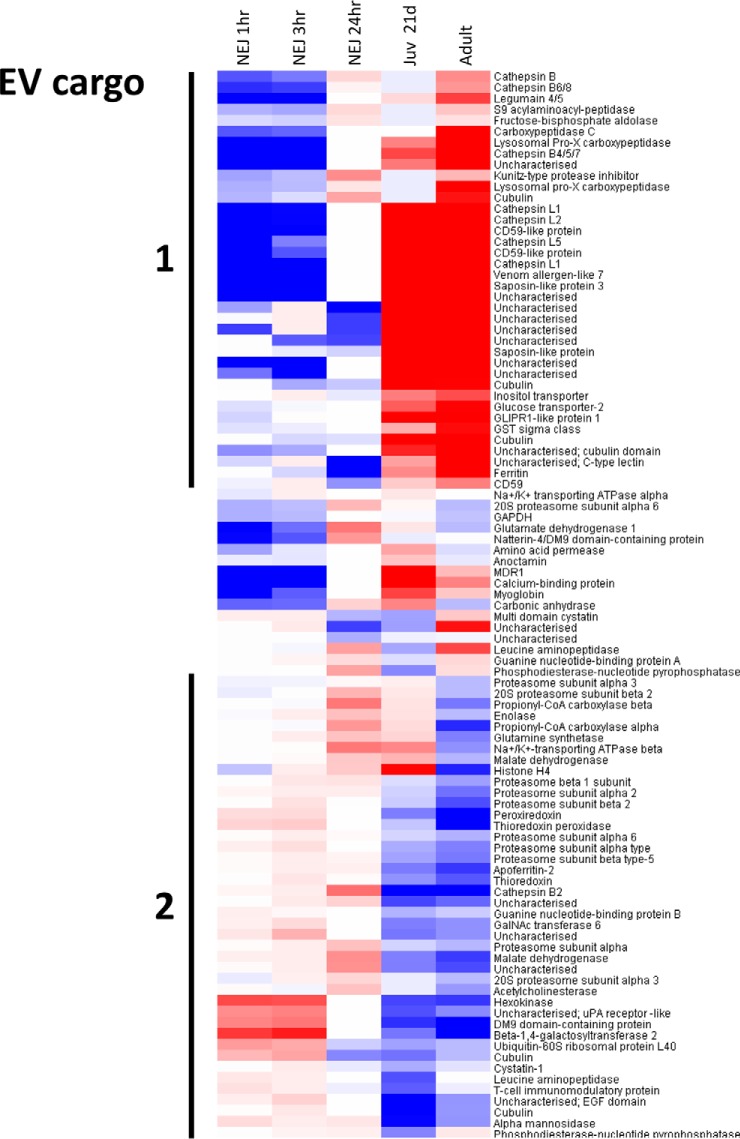
**Expression of transcripts encoding putative EV cargo proteins across the intramammalian life cycle stages of *F. hepatica***. Heatmaps are colored on a log_2_ scale with up-regulation in red and down-regulation in blue relative to metacercariae, depicting log fold change between developmental stages, grouped by hierarchical clustering. The *F. hepatica* life cycle stages analyzed are: newly excysted juveniles (NEJ; 1-, 3- and 24-h postexcystment), 21-day-old immature liver-stage flukes (Juv_21d), and adult flukes from the bile duct.

Having identified putative homologs of the EV biogenesis pathway members in *F. hepatica*, we then examined their relative expression levels across the intramammalian lifecycle stages of *F. hepatica.* Transcriptome data sets for *Fasciola* NEJs at 1 h, 3 h, and 24 h time-points postexcystment were used as well as 21-day old immature liver-stage flukes and adult flukes from the bile duct (11). Relative transcript expression levels (fold change on a log_2_ scale) are shown as a heat-map in Fig. 5. Taking this broad view, it is apparent that, with a few exceptions, putative EV biogenesis pathway members in *F. hepatica* are constitutively expressed (albeit at low levels), and do not differ greatly in expression levels as the parasite migrates from the small intestine, through the liver and into the bile ducts. However, when we performed the same analysis of the transcripts encoding the remaining proteins identified during our proteomics analysis of adult fluke exosome-like EVs (*i.e.* those not implicated in EV biogenesis and thus termed putative cargo molecules), we found that most were expressed at higher levels and were developmentally regulated. As can be seen in the heat-map shown in Fig. 6, the transcripts can be broadly segregated into two groups: (1) those with low levels of expression in the NEJ stage that increase in the 21-day old and adult flukes, and (2) those with high levels of expression in the NEJ stage that decrease in the 21-day old and adult flukes. Group one members included several proteases such as legumain 4/5, Pro-Xaa carboxypeptidase, cathepsin B4/5/7 and members of cathepsin L clades 1, 2, and 5. Others included saposin-like and CD59-like proteins, a calcium-binding protein, and a range of highly expressed, but uncharacterized, proteins. Notable group two members included thioredoxin, peroxiredoxin, cathepsin B2, various proteasome subunits and metabolic enzymes such as enolase, malate dehydrogenase, and propionyl CoA carboxylase.

## DISCUSSION

The recent availability of new -omics resources for *F. hepatica* permitted us to conduct the most comprehensive analysis of any helminth secretome to date. We have discovered that adult *F. hepatica* secretions are more complex than previously thought, and contain at least two subpopulations of EVs that differ according to size, cargo and mechanism of release from the parasite. In doing so we have challenged the classic view that soluble proteins are the sole mediators of the host-parasite interaction and identified new mechanisms by which parasite-derived molecules can interact with host cells. These mechanisms can be classed under various headings: (1) *Extracellular interaction with host macromolecules*. In terms of protein content, soluble molecules made the greatest contribution to the adult fluke total secretome (87.8%). This fraction is dominated by members of the cathepsin L cysteine peptidase family which represent >80% of the total protein secreted (8–10). *Fasciola* cathepsin Ls perform a variety of roles, mostly associated with extracellular degradation of host macromolecules such as collagen to aid parasite migration through host tissues (36, 37), hemoglobin as a source of nutrition (30) and immunoglobulins to avoid expulsion by the host immune system (40). The considerable expenditure devoted to secretion of soluble proteins by *F. hepatica* clearly shows that these tasks are critical for completion of the parasite's life-cycle. (2) *Endocytosis of soluble parasite molecules*. We have shown that cathepsin L1 and helminth defense molecule (HDM), which are both present as soluble proteins within *F. hepatica* secretions, are internalized via the endolysosomal pathway and modulate host macrophage function by degradation of toll-like receptor 3 and inhibition of vacuolar-ATPase respectively (26, 41). The receptors required for uptake of these molecules are currently unknown however HDM binds to cholesterol within lipid rafts on the cell surface that may initiate internalization (26). (3) *Internalization of parasite EVs*. Although we estimate that the 15 K and 120 K EV pellets hold only 8.6% and 3.6% of the total protein secreted by adult *F. hepatica*, respectively, they contain potent immunomodulators/virulence factors (see below) that would exert greater effects on host cell function than their abundance might suggest. EVs secreted by adult flukes will drain, via the bile ducts, into the host gut and we have shown that fluke exosome-like EVs are internalized by host intestinal epithelial cells (IECs) *in vitro* (12). Although traditionally regarded as a mere physical barrier to infection, IECs are now known to express pattern-recognition receptors that enable them to detect a range of microbial ligands and transmit signals to underlying gut-associated lymphoid tissues (42). Thus, parasite-derived EVs could maintain a systemic Th2/regulatory environment that is permissive to fluke survival and reproduction via modulation of IEC immune surveillance.

We, and others, have shown that several proteins released by *F. hepatica* have the ability to modulate host immune cell function (43). In this study we have found the majority of these immunomodulators are packaged within the exosome-like EVs secreted by adult fluke. Although *F. hepatica* peroxiredoxin (Prx) actively drives host Th2 immune responses via stimulation of M2 macrophages (44, 45), most of the other fluke immunomodulators identified so far (despite displaying varied structures and biochemical activities), indirectly promote Th2 responses by suppressing the development of Th1/Th17-associated inflammation. These include *Fasciola* helminth defense molecule (HDM), cathepsin L1 (FhCL1), fatty acid-binding protein (FABP), kunitz-type molecule (Fh-KTM) and Sigma class glutathione transferase (GST) (23, 41, 46–48). The role of *F. hepatica* Prx in switching the host immune response to a Th2 phenotype during the early stages of infection has been well documented (44, 45). This is in agreement with our transcriptome profiling of EV cargo molecules (Group 2, Fig. 6). However, our transcriptomics and proteomics profiling experiments have shown that the remaining immunomodulators (FhCL1, HDM, FABP, Fh-KTM, and GST sigma) are constitutively expressed across the intramammalian life-cycle stages of the fluke and are actually up-regulated as the flukes migrate through the liver, enter the bile ducts and mature into adult worms (10,11; Group 1, Fig. 6). These observations suggest that the latter molecules act to maintain the Th2/regulatory phenotype seen during chronic infection. Thus, EV cargo molecules, as well as components of the soluble secretome of adult *F. hepatica* (10) are developmentally regulated to facilitate the parasites migration through host tissue and to counteract host immune attack. Work is ongoing in our laboratory to characterize the EVs secreted by the NEJ stage.

We have also identified potential immunomodulators not previously thought to be secreted by adult *F. hepatica*, the most notable being members of the cathepsin B family. Several cathepsin B transcripts were previously identified in adult flukes, but these enzymes were not detected in the adult fluke secretome and therefore it was suggested that they have intracellular house-keeping roles (10). However, we have found that the small exosome-like EVs contain at least four developmentally regulated cathepsin B peptidases. This finding establishes that both cathepsin L and B peptidases are secreted by adult flukes, albeit via different mechanisms, the former via the gut and the latter via the tegument. Studies in *Schistosoma mansoni* have shown that a cathepsin B, SmCB2, is located in the tegument of the adult parasites (49). Also identified, within the EV lumen, were a number of leaderless proteins (including Prx, heat shock proteins and annexins) that are homologs of host damage-associated molecular pattern molecules (DAMPs). DAMPs are leaderless cytosolic proteins that are released into the extracellular environment via atypical secretory pathways in response to tissue damage (50). They interact with pattern recognition receptors expressed by immune cells and thus set off an “alarm” system which often leads to a wound-healing scenario (50). Because of their abundance within helminth secretions, we previously speculated that DAMP homologs can direct the host immune response (51) and their packaging into EVs provides an alternative route for their non-canonical secretion. Although the mechanism for targeting cytosolic proteins into EVs is not fully understood, ATP-binding cassette (ABC) transporters are responsible for packaging some leaderless proteins (*e.g.* FGF1, IL-1β, and Hsp70) into EVs destined for secretion (52–54). We identified two ABC transporters associated with the *F. hepatica* EV membrane (ABCB1 and p-glycoprotein 1) that may perform similar packaging roles in the parasite.

The recently available *F. hepatica* genome (11) has allowed us, for the first time, to identify pathways involved in EV biogenesis in a helminth parasite. Exosomes arise from the endosomal system and are formed by inward budding of the multi-vesicular body (MVB) membrane that allows capture of cytoplasmic cargo. This step is thought to occur via two different routes. Endosomal sorting complexes required for transport (ESCRT) 0, I, II, and III, together with syntenin-ALIX complexes, capture ubiquitinated proteins, whereas in the ESCRT-independent pathway tetraspanins (*e.g.* CD63) and various lipid-related enzymes (notably sphingomyelinase) can also drive vesicle formation (55, 56). The final abscission of the budding exosomes into the MVB lumen requires the AAA-ATPase VPS4 (57). The exosomes are then secreted into the extracellular space following the fusion of the MVB with the plasma membrane, which is dependent on small GTPases including Rab8b, Rab11, Rab27a, and Rab27b and the Rab effectors otoferlin and synaptotagmin-like protein (58–61). We identified *F. hepatica* homologs of all these key regulators of exosome biogenesis during our proteomics analysis of the 120 K EV pellet (summarized in Fig. 4). However, it is significant that although the ESCRT pathway was well represented at the proteome level (10 members were identified in the MS analysis), the only representatives of the lipid-related/ESCRT-independent pathway were sphingomyelinase and tetraspanin CD63 (supplemental Table S4). Based on these findings we propose that exosome biogenesis in adult fluke primarily occurs via ESCRT-dependent MVB formation in the tegumental syncytium with the exosomes being shed from the apical plasma membrane (although functional studies such as RNAi are needed to confirm this). In support of this proposal, MVBs have been described during ultrastructural examination of the tegumental syncytia of *F. hepatica* and the related trematode *E. caproni* (6, 12). Furthermore, our comparative proteomics analysis revealed that 42% of the proteins identified in the *F. hepatica* tegumental extracts by Wilson *et al.* (6) were also packaged into the 120 K EVs; these contained a variety of proteins (*e.g.* tegumental antigen 1, DM9-containing protein, annexin, and tetraspanin) that localize primarily within the tegument of *F. hepatica* and related trematodes (6, 38, 62–64). Determining the specific roles of individual pathway members during exosome biogenesis in liver fluke needs further investigation, perhaps by RNAi-mediated gene silencing which is functional in this parasite (65).

Targeting key regulators of EV biogenesis (including sphingomyelinase, ALIX and Rab GTPases) using chemical inhibitors or RNAi has been shown to significantly reduce the number of EVs released by mammalian cells. Indeed, suppressing EV production to inhibit their effect in disease is an emerging therapeutic strategy that has yielded impressive results, notably in cancer therapy (reviewed by 14). We have identified *F. hepatica* homologs of several key regulators of EV biogenesis that could be targeted for parasite control if specific inhibitors can be found. Although technically challenging, selective inhibition of parasite EV biogenesis would prevent the delivery of a range of immunomodulators to host cells and, conceivably, tip the host immune balance in favor of Th1-driven parasite elimination. An alternative anti-parasite control strategy would be to block the uptake of parasite-derived EVs by host cells. Although direct fusion of EVs with the plasma membrane of target cells has been reported (66), uptake mechanisms akin to receptor-mediated endocytosis (dependent on the complement of proteins expressed on the vesicle surface) appear to be the dominant route of entry into recipient cells (67). Many of the proteins we identified on the external surface of *F. hepatica* EVs are metabolic enzymes, or other housekeeping proteins, with reported “moonlighting” roles in other helminths and microbial pathogens (68). These include malic enzyme, enolase, GAPDH, aldolase and annexin which act as parasite adhesion molecules via interaction with plasminogen and/or fibronectin on the host cell surface (69–74) and could be targets for blocking antibodies. Recently, it was shown that antibodies against *O. viverrini* tetraspanin blocked uptake of parasite EVs by host cholangiocytes, providing proof-of-concept, at least mechanistically, for this approach as an anti-parasite therapy (75).

In this report we have performed the first comparative analysis of the soluble and vesicular secretions of a helminth parasite. This was made possible by integrating the recently available genome, transcriptome, and proteome data sets for this pathogen (11). Our study underscores the importance of investigating the role of EVs during acute and chronic fluke infection and presents new opportunities to disrupt EV biogenesis or interaction with target cells toward parasite control.

## Supplementary Material

Supplemental Data

## Footnotes

^1^ The abbreviations used are:

EV: extracellular vesicle
NEJ: newly excysted juvenile
HDM: helminth defence molecule
CB: cathepsin B
CL: cathepsin L
LAP: leucine aminopeptidase
Prx: peroxiredoxin
FaBP: fatty acid-binding protein
GST: glutathione transferase
MVB: multivesicular body
ESCRT: endosomal sorting complex required for transport
DAMP: damage-associated molecular pattern.
