# Supplementary material for: The Extracellular Vesicles of the Helminth Pathogen, Fasciola hepatica: Biogenesis Pathways and Cargo Molecules Involved in Parasite Pathogenesis
Source: Mol Cell Proteomics. 2015 Oct 20;14(12):3258–73. doi: 10.1074/mcp.M115.053934 (PMC4762619; doi:10.1074/mcp.M115.053934)
Supplement: Supplemental Data [file supp_14_12_3258__index.html]

The extracellular vesicles of the helminth pathogen, Fasciola hepatica: biogenesis pathways and cargo molecules involved in parasite pathogenesis. — The Extracellular Vesicles of the Helminth Pathogen, Fasciola hepatica: Biogenesis Pathways and Cargo Molecules Involved in Parasite Pathogenesis — The Extracellular Vesicles of Fasciola hepatica — Supplemental Data 

# The Extracellular Vesicles of the Helminth Pathogen, *Fasciola hepatica*: Biogenesis Pathways and Cargo Molecules Involved in Parasite Pathogenesis

## Supplemental Data

- Supplementary Table 4 (.docx, 37 KB) - Putative homologs of the EV biogenesis pathway members in F. hepatica
- Supplementary figure 1 (.pdf, 490 KB) - Transmission electron microscopy of the exosome-like 120K vesicle pellet (A) and the 15K vesicle pellet (B-E).
- Supplementary figure 2 (.pdf, 284 KB) - Proteins identified in the exosome-like EVs and total secretome of adult F. hepatica grouped according to function.
- Supplementary Table 1 (.docx, 18 KB) - Proteins identified from the total secretome of adult F. hepatica grouped according to function.
- Supplementary Table 2 (.xls, 38 KB) - Identification of proteins within the soluble secretome of adult F. hepatica by LC-MS/MS.
- Supplementary Table 3 (.xls, 68 KB) - LC-MS/MS identification of proteins in different fractions of extracellular vesicles released by adult F. hepatica.
