# Supplementary material for: The Extracellular Vesicles of the Helminth Pathogen, Fasciola hepatica: Biogenesis Pathways and Cargo Molecules Involved in Parasite Pathogenesis
Source: Mol Cell Proteomics. 2015 Oct 20;14(12):3258–73. doi: 10.1074/mcp.M115.053934 (PMC4762619; doi:10.1074/mcp.M115.053934)
Supplement: Supplemental Data [file 10.1074_M115.053934_mcp.M115.053934-1.docx]

**Supplementary Table 4:** Putative homologs of the EV biogenesis pathway members in F. hepatica

Human proteins, inferred from the literature to have roles in exosome biogenesis, cargo sorting or uptake, were used as BLAST queries to identify homologs in closely-related trematode species with well annotated genomes. Both the human proteins and trematode homologs were used as BLAST queries to interrogate the *F. hepatica* genome. ^1^Trematode or human (in brackets) query sequence. Those used to interrogate the *F. hepatica* genome are shown in bold; ^2^L = lumen, M = membrane-associated.

| **Protein name** | **Uniprot accession^1^** | ***F. hepatica* identifier** | **E Value** | **Score (bits)** | **Identities (%)** | **Positives (%)** | **Gaps (%)** | **MS matches^2^** |
| --- | --- | --- | --- | --- | --- | --- | --- | --- |
| Components of the ESCRT dependent pathway | | | | | | | | |
| **ESCRT-0** |  | |  |  |  |  |  |  |
| HGS | [**H2KVA9**](http://www.uniprot.org/uniprot/H2KVA9) (O14964) | BN1106_s4722B000062 | 1e-111 | 338 | 68 | 77 | 4 |  |
| STAMBP | [**H2KPP3**](http://www.uniprot.org/uniprot/H2KPP3) (O95630) | BN1106_s2325B000322 | 2e-126 | 374 | 48 | 64 | 7 |  |
| STAM | [**G7Y9K6**](http://www.uniprot.org/uniprot/G7Y9K6) (Q92783) | BN1106_s7731B000034 | 1e-178 | 512 | 63 | 71 | 13 |  |
| **ESCRT-I** |  |  |  |  |  |  |  |  |
| TSG101 | [**H2KP02**](http://www.uniprot.org/uniprot/H2KP02) (Q99816) | BN1106_s410B000432 | 2e-052 | 180 | 39 | 51 | 16 |  |
| VPS28* | [**Q5DGV7**](http://www.uniprot.org/uniprot/Q5DGV7) (Q9UK41) | BN1106_s3801B000106 | 1e-038 | 133 | 58 | 67 | 25 |  |
| VPS37 | [**G7YTY4**](http://www.uniprot.org/uniprot/G7YTY4) (Q8NEZ2) | BN1106_s6094B000080 | 1e-014 | 67 | 44 | 61 | 0 |  |
| MVB12a | [**H2KR27**](http://www.uniprot.org/uniprot/H2KR27) (Q96EY5) | BN1106_s335B000432 | 6e-012 | 61.6 | 41 | 61 | 5 |  |
| **ESCRT-II** |  |  |  |  |  |  |  |  |
| VPS22/SNF8 | [**G7Y7B2**](http://www.uniprot.org/uniprot/G7Y7B2) (Q96H20) | BN1106_s3610B000067 | 3e-063 | 198 | 65 | 77 | 0 |  |
| VPS25 | [**G7YNR9**](http://www.uniprot.org/uniprot/G7YNR9) (Q9BRG1) | BN1106_s8922B000034 | 2e-096 | 303 | 71 | 88 | 0 |  |
| VPS36 | [**H2KUN0**](http://www.uniprot.org/uniprot/H2KUN0) (Q86VN1) | BN1106_s1285B000160 | 8e-188 | 352 | 50 | 64 | 11 |  |
| **ESCRT-III** |  |  |  |  |  |  |  |  |
| CHMP2A | [**G7YBN0**](http://www.uniprot.org/uniprot/G7YBN0) (O43633) | BN1106_s912B000169 | 5e-095 | 291 | 74 | 83 | 3 | L |
| CHMP2B | [**G7YMJ9**](http://www.uniprot.org/uniprot/G7YMJ9) (Q9UQN3) | BN1106_s3344B000075 | 2e-063 | 214 | 76 | 81 | 10 |  |
| CHMP6 | [**H2KSF3**](http://www.uniprot.org/uniprot/H2KSF3) (Q96FZ7) | No match | - | - | - | - | - |  |
| CHMP3 | [**H2KVS7**](http://www.uniprot.org/uniprot/H2KVS7) (Q9Y3E7) | BN1106_s2567B000083 | 6e-067 | 208 | 56 | 71 | 1 |  |
| CHMP4 | [**H2KVP6**](http://www.uniprot.org/uniprot/H2KVP6) (Q9BY43) | BN1106_s2597B000195 | 2e-071 | 223 | 64 | 82 | 0 |  |
| CHMP5 | [**G7YQI3**](http://www.uniprot.org/uniprot/G7YQI3) (Q9NZZ3) | BN1106_s6543B000070 | 1e-107 | 312 | 72 | 83 | 2 | L/M |
| CHMP1a | [**Q9HD42**](http://www.uniprot.org/uniprot/Q9HD42) (Q9HD42) | BN1106_s2655B000264 | 2e-059 | 187 | 48 | 73 | 2 | L/M |
| CHMP1b | [**Q7LBR1**](http://www.uniprot.org/uniprot/Q7LBR1) (Q7LBR1) | BN1106_s2316B000077 | 3e-079 | 238 | 62 | 79 | 1 | L/M |
| IST1 | [**G7YUN3**](http://www.uniprot.org/uniprot/G7YUN3) (P53990) | BN1106_s3747B000112 | 1e-096 | 306 | 54 | 70 | 3 | L/M |
| **Vps4-Vta1 complex** |  | |  |  |  |  |  |  |
| VPS4 | [**H2KR36**](http://www.uniprot.org/uniprot/H2KR36) (Q9UN37) | BN1106_s1437B000141 | 0.0 | 555 | 82 | 91 | 0 | L/M |
| VTA1 | [**G7YED6**](http://www.uniprot.org/uniprot/G7YED6) (Q9NP79) | BN1106_s2858B000111 | 4e-070 | 222 | 60 | 70 | 8 | L/M |
| **Bro1/ALIX** |  | |  |  |  |  |  |  |
| ALIX | [**H2KNH6**](http://www.uniprot.org/uniprot/H2KNH6) (Q8WUM4) | BN1106_s1871B000313 | 0.0 | 759 | 58 | 73 | 6 | L/M |
| BRO1 domain-containing protein (ALIX) | **H2KNG3** | BN1106_s2963B000136 | 1e-173 | 497 | 57 | 76 | 3 | L/M |
| **Related components** |  | |  |  |  |  |  |  |
| Syndecan | [G7YEG1](http://www.uniprot.org/uniprot/G7YEG1) (P18827) | BN1106_s6866B000040 | 1e-065 | 212 | 51 | 69 | 7 |  |
| Syntenin 1 | [**G7Y6Z7**](http://www.uniprot.org/uniprot/G7Y6Z7) (O00560) | BN1106_s4740B000062 | 2e-051 | 172 | 36 | 55 | 5 | L/M |
| SIMPLE | [**Q99732**](http://www.uniprot.org/uniprot/Q99732) | BN1106_s3217B000130 | 8e-018 | 76.6 | 48 | 65 | 0 |  |
|  |  |  |  |  |  |  |  |  |
| Components of the ESCRT independent pathways | | | | | | | | |
| - **Ceramide and lipids pathway** | | | | | | | | |
| **Lipolytic enzymes** | | | | | | | | |
| SMPD2, Neutral sphingomyelinase | [**O60906**](http://www.uniprot.org/uniprot/O60906) | BN1106_s7135B000046 | 2e-048 | 172 | 34 | 54 | 7 |  |
| Acid sphingomyelinase | **H2KTZ7** (P17405) | BN1106_s3568B000138 | 9e-131 | 394 | 40 | 59 | 6 | L/M |
| SMS2,Sphingomyelin synthase 2 | [**H2KQF2**](http://www.uniprot.org/uniprot/H2KQF2) (Q8NHU3) | BN1106_s3939B000104 | 1e-157 | 460 | 62 | 73 | 5 |  |
| SphK2, Sphingosine kinase 2 | [**C1LJ82**](http://www.uniprot.org/uniprot/C1LJ82) (Q9NRA0) | No match | - | - | - | - | - |  |
| PLD, Phospholipase D | [**G7YNY2**](http://www.uniprot.org/uniprot/G7YNY2) (O14939) | BN1106_s3211B000083 | 5e-139 | 429 | 58 | 74 | 1 |  |
| PLA2, phospholipase A2 | [**G7YM87**](http://www.uniprot.org/uniprot/G7YM87) (P04054) | BN1106_s1517B000259 | 0.0 | 578 | 46 | 60 | 11 |  |
| Phospholipase B-like 2 | **C1LID9** (Q8NHP8) | BN1106_s1597B000141 | 6e-104 | 326 | 47 | 64 | 1 | L |
| **Signal transduction** | | | | | | | | |
| Flotillin 1 | [**C1LLP3**](http://www.uniprot.org/uniprot/C1LLP3) (O75955) | BN1106_s517B000379 | 0.0 | 708 | 82 | 92 | 0 |  |
| Flotillin 2 | [**C7TZR5**](http://www.uniprot.org/uniprot/C7TZR5) (Q14254) | BN1106_s456B000241 | 0.0 | 629 | 66 | 78 | 14 |  |
| DKG | [**G7YB49**](http://www.uniprot.org/uniprot/G7YB49) (P23743) | BN1106_s667B000211 | 0.0 | 1439 | 72 | 78 | 9 |  |
| **Lipids transport** | | | | | | | | |
| ABCA1 | [**G7YFR4**](http://www.uniprot.org/uniprot/G7YFR4) (O95477) | BN1106_s1525B000204 | 0.0 | 711 | 53 | 67 | 6 |  |
| ABCA3 | **H2KVB6** (Q99758) | BN1106_s226B000394 | 1e-131 | 425 | 68 | 80 | 0 |  |
| ABCB1 | ([**P08183**](http://www.uniprot.org/uniprot/P08183)**)** | BN1106_s634B000589 | 0.0 | 892 | 39 | 58 | 4 | L/M |
| MDR1/P-gp | **G4VIC6** | BN1106_s2471B000098 | 0.0 | 1259 | 51 | 68 | 2 | L/M |
| Oligosaccharidyl-lipid flippase family | [**H2KUI4**](http://www.uniprot.org/uniprot/H2KUI4) | BN1106_s99B000255 | 0.0 | 625 | 58 | 72 | 8 |  |
| Flippase | **G7YEB4** | BN1106_s435B000242 | 0.0 | 737 | 80 | 87 | 3 | L/M |
| Phospholipid scramblase 2 | [**G7YAS3**](http://www.uniprot.org/uniprot/G7YAS3) (Q9NRY7) | BN1106_s1682B000370 | 2e-075 | 234 | 61 | 81 | 0 |  |
| Phospholipid scramblase 3 | [**G7YL60**](http://www.uniprot.org/uniprot/G7YL60) (Q9NRY6) | BN1106_s848B000726 | 3e-041 | 138 | 51 | 69 | 1 |  |
| Oxysterol binding protein | [**G7YVH1**](http://www.uniprot.org/uniprot/G7YVH1) (P22059) | BN1106_s2635B000230 | 0.0 | 652 | 65 | 77 | 4 |  |
| Niemann-Pick C1 protein | **G7YQQ4** (O15118) | BN1106_s1498B000257 | 0.0 | 991 | 56 | 69 | 7 | L |
| Niemann-Pick C2 protein | **G7YJT4** (P61916) | BN1106_s20469B000004 | 1e-049 | 139 | 43 | 64 | 2 | L/M |
|  |  |  |  |  |  |  |  |  |
| - **Tetraspanins** | | |  |  |  |  |  |  |
| CD63 antigen | [**G7YRI5**](http://www.uniprot.org/uniprot/G7YRI5) (P08962) | BN1106_s4560B000072 | 1e-068 | 228 | 62 | 74 | 1 |  |
| Tetraspanin CD63-receptor | [**H2KVE5**](http://www.uniprot.org/uniprot/H2KVE5) | BN1106_s1657B000161 | 6e-037 | 135 | 30 | 51 | 10 | L/M |
| CD9 antigen | [**G7YQ13**](http://www.uniprot.org/uniprot/G7YQ13) (P21926) | No match | - | - | - | - | - |  |
| Tetraspanin 1 | [**G7Y810**](http://www.uniprot.org/uniprot/G7Y810) (O60635) | BN1106_s915B000136 | 5e-118 | 345 | 54 | 71 | 0 | L/M |
| CD81 | [**G7YAH0**](http://www.uniprot.org/uniprot/G7YAH0) (P60033) | BN1106_s4022B000148 | 7e-065 | 203 | 73 | 89 | 0 |  |
| Tspan8 | **(P19075)** | No match | - | - | - | - | - |  |
| CD37 | ([**P11049**](http://www.uniprot.org/uniprot/P11049)**)** | No match | - | - | - | - | - |  |
| CD82 | **(P27701)** | No match | - | - | - | - | - |  |
| CD151 | **(P48509)** | No match | - | - | - | - | - |  |
|  |  |  |  |  |  |  |  |  |
| Cargo sorting | | |  |  |  |  |  |  |
| **Protein sorting** |  | |  |  |  |  |  |  |
| Heat shock protein 70 | [**B1NI98**](http://www.uniprot.org/uniprot/B1NI98) (P0DMV8) | BN1106_s309B000234 | 0.0 | 1077 | 84 | 92 | 0 | L/M |
| Hsp90 (alpha) | ([**P07900**](http://www.uniprot.org/uniprot/P07900)**)** | BN1106_s1320B000236 | 0.0 | 806 | 75 | 88 | 2 |  |
| 14-3-3 | [**H2KNZ3**](http://www.uniprot.org/uniprot/H2KNZ3) | BN1106_s686B000273 | 2e-140 | 398 | 80 | 86 | 0 |  |
| 14-3-3 protein β/α-1 | **Q5FX78** | BN1106_s3904B000042 | 0.0 | 520 | 99 | 99 | 0 | L |
| **RNA sorting** |  | |  |  |  |  |  |  |
| hnRNPA2B1 | [**H2KT13**](http://www.uniprot.org/uniprot/H2KT13) (P22626) | BN1106_s3553B000143 | 1e-173 | 486 | 81 | 87 | 6 |  |
| Annexin B2/Annex 7 | [**C3VEV0**](http://www.uniprot.org/uniprot/C3VEV0) | BN1106_s500B000161 | 8e-065 | 211 | 38 | 56 | 19 | L/M |
| Major vault protein | **G4V9U9** | BN1106_s7273B000042 | 0.0 | 1179 | 66 | 82 | 1 | L/M |
| **Lipid sorting** | | | | | | | | |
| Leukotriene-A4 hydrolase | **(P09960 )** | BN1106_s98B000759 | 1e-125 | 384 | 42 | 60 | 11 |  |
| Prostaglandin E synthase 3 | [**G7YDD8**](http://www.uniprot.org/uniprot/G7YDD8) (Q9H7Z7) | BN1106_s2740B000079 | 1e-036 | 125 | 74 | 79 | 7 |  |
|  |  |  |  |  |  |  |  |  |
| Membrane trafficking and cytoskeleton regulation | | |  |  |  |  |  |  |
| **Small GTPases** |  | |  |  |  |  |  |  |
| RAB27A | [**H2KNW4**](http://www.uniprot.org/uniprot/H2KNW4) (P51159) | BN1106_s1792B000145 | 3e-160 | 446 | 93 | 98 | 0 | L |
| RAB35 | [**G7Y8S9**](http://www.uniprot.org/uniprot/G7Y8S9) (Q15286) | BN1106_s2985B000113 | 1e-046 | 161 | 87 | 93 | 0 |  |
| Rab-protein 11 | [**C1L612**](http://www.uniprot.org/uniprot/C1L612) (P62491) | BN1106_s844B000259 | 5e-102 | 298 | 66 | 77 | 8 | L/M |
| Rab-8A | [**Q86ET1**](http://www.uniprot.org/uniprot/Q86ET1) (P61006) | BN1106_s258B000276 | 2e-074 | 225 | 61 | 79 | 0 | L/M |
| Ras-related protein Ral-A | **G7YCE6** | BN1106_s637B000246 | 2e-055 | 187 | 68 | 82 | 0 | L/M |
| ARF6 | ([**P62330**](http://www.uniprot.org/uniprot/P62330)**)** | BN1106_s2303B000142 | 2e-104 | 300 | 79 | 91 | 1 |  |
| Ras-like GTP-binding protein Rho1 | **S4PGT9** | BN1106_s1908B000177 | 9e-122 | 346 | 88 | 94 | 1 | L/M |
| TBC1 domain family member 20 | **H2KNS4** | BN1106_s92B000559 | 2e-105 | 335 | 50 | 66 | 7 | L |
| **SNAREs** |  | |  |  |  |  |  |  |
| Syntaxin | [**G7YP44**](http://www.uniprot.org/uniprot/G7YP44) (Q16623) | BN1106_s238B000371 | 4e-042 | 145 | 72 | 77 | 22 |  |
| Synaptobrevin homolog YKT6 | [**H2KTY8**](http://www.uniprot.org/uniprot/H2KTY8) (O15498) | BN1106_s5169B000151 | 3e-050 | 171 | 87 | 91 | 0 |  |
| Synaptotagmin | **H2KPI2** (P21579) | BN1106_s3353B000056 | 4e-143 | 415 | 52 | 71 | 1 | L |
| Synaptosomal-associated protein | [**H2KRN3**](http://www.uniprot.org/uniprot/H2KRN3) | BN1106_s3959B000138 | 8e-133 | 376 | 85 | 89 | 6 |  |
| VAMP7 | [**G7YB99**](http://www.uniprot.org/uniprot/G7YB99) (P51809) | BN1106_s171B000376 | 1e-046 | 154 | 52 | 70 | 0 | L |
| **Proton pumps** |  | |  |  |  |  |  |  |
| V-type H+-transporting ATPase subunit A | [**H2KPW7**](http://www.uniprot.org/uniprot/H2KPW7) | BN1106_s2110B000156 | 0.0 | 1058 | 85 | 89 | 7 |  |
| V-ATPase, VHA5 | [**G5EEK9**](http://www.uniprot.org/uniprot/G5EEK9) | BN1106_s4862B000066 | 1e-163 | 494 | 43 | 61 | 13 | L |
| H+-transporting ATPase | **H2KTS1** | BN1106_s18772B000008 | 2e-099 | 311 | 75 | 80 | 6 | L |
| ATPase, H+ transporting, lysosomal accessory protein 1 | **C1LMZ1** (P11279) | BN1106_s3321B000106 | 2e-048 | 160 | 42 | 62 | 2 | L |
| Vacuolar H+ ATPase | **C1LDA3** | BN1106_s2593B000233 | 8e-145 | 414 | 71 | 77 | 12 | L |
| **Cytoskeleton regulation, adhesion, membrane fusion and repair** | | | | | | | | |
| Calpain | [**P27730**](http://www.uniprot.org/uniprot/P27730) | BN1106_s204B000249 | 0.0 | 1058 | 65 | 80 | 1 |  |
| Gelsolin | [**C1LDA7**](http://www.uniprot.org/uniprot/C1LDA7) (P06396) | BN1106_s2349B000191 | 2e-147 | 427 | 62 | 77 | 0 | L/M |
| Myosin light chain kinase | [**H2KUH9**](http://www.uniprot.org/uniprot/H2KUH9) (Q32MK0) | BN1106_s398B000242 | 0.0 | 575 | 82 | 90 | 1 |  |
| ERK, Extracellular signal-regulated kinase | [**H2KSY3**](http://www.uniprot.org/uniprot/H2KSY3) (P27361) | BN1106_s2009B000121 | 1e-069 | 236 | 42 | 60 | 6 |  |
| Annexin B22 | [**C4QH88**](http://www.uniprot.org/uniprot/C4QH88) | BN1106_s819B000365 | 1e-126 | 369 | 65 | 82 | 3 | L/M |
| Annexin | **G7Y5I1** | BN1106_s945B000218 | 3e-155 | 465 | 54 | 63 | 14 | L/M |
| Annexin | **H2KP46** | BN1106_s3266B000046 | 2e-176 | 510 | 54 | 67 | 12 | L |
| Thrombospondin 2 | [**H2KTG5**](http://www.uniprot.org/uniprot/H2KTG5) (P35442) | BN1106_s3238B000040 | 2e-152 | 460 | 53 | 71 | 0 |  |
| Vesicle-fusing ATPase | [**G7Y509**](http://www.uniprot.org/uniprot/G7Y509) | BN1106_s908B000155 | 0.0 | 1236 | 85 | 93 | 1 |  |
| Myoferlin | [**G7YHB9**](http://www.uniprot.org/uniprot/G7YHB9) (Q9NZM1) | BN1106_s3585B000136 | 0.0 | 1013 | 64 | 77 | 1 | L/M |
| Otoferlin | [**G7YR21**](http://www.uniprot.org/uniprot/G7YR21) (Q9HC10) | BN1106_s3261B000048 | 0.0 | 1509 | 67 | 83 | 1 | L/M |
|  |  |  |  |  |  |  |  |  |
