# Supplementary material for: The Extracellular Vesicles of the Helminth Pathogen, Fasciola hepatica: Biogenesis Pathways and Cargo Molecules Involved in Parasite Pathogenesis
Source: Mol Cell Proteomics. 2015 Oct 20;14(12):3258–73. doi: 10.1074/mcp.M115.053934 (PMC4762619; doi:10.1074/mcp.M115.053934)

Supplementary Figure 1

Transmission electron microscopy of the exosome-like 120K vesicle pellet (A) and the 15K vesicle pellet (B-E).

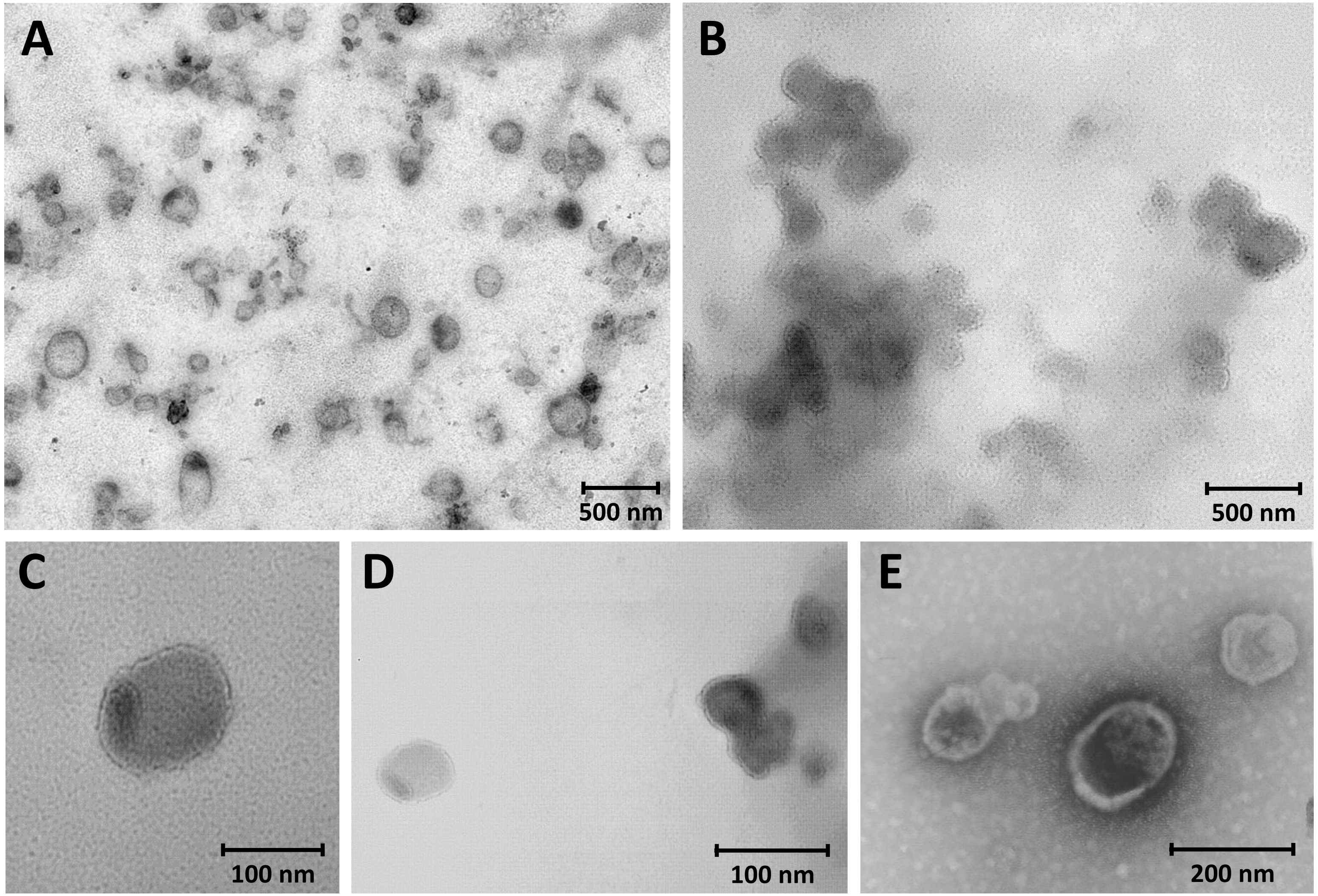

Supplement: Supplemental Data [file 10.1074_M115.053934_mcp.M115.053934-2.pdf]
