# Supplementary material for: The Extracellular Vesicles of the Helminth Pathogen, Fasciola hepatica: Biogenesis Pathways and Cargo Molecules Involved in Parasite Pathogenesis
Source: Mol Cell Proteomics. 2015 Oct 20;14(12):3258–73. doi: 10.1074/mcp.M115.053934 (PMC4762619; doi:10.1074/mcp.M115.053934)

Supplementary Figure 2
Proteins identified in the exosome-like EVs and total secretome of adult *F. hepatica* grouped according to function.

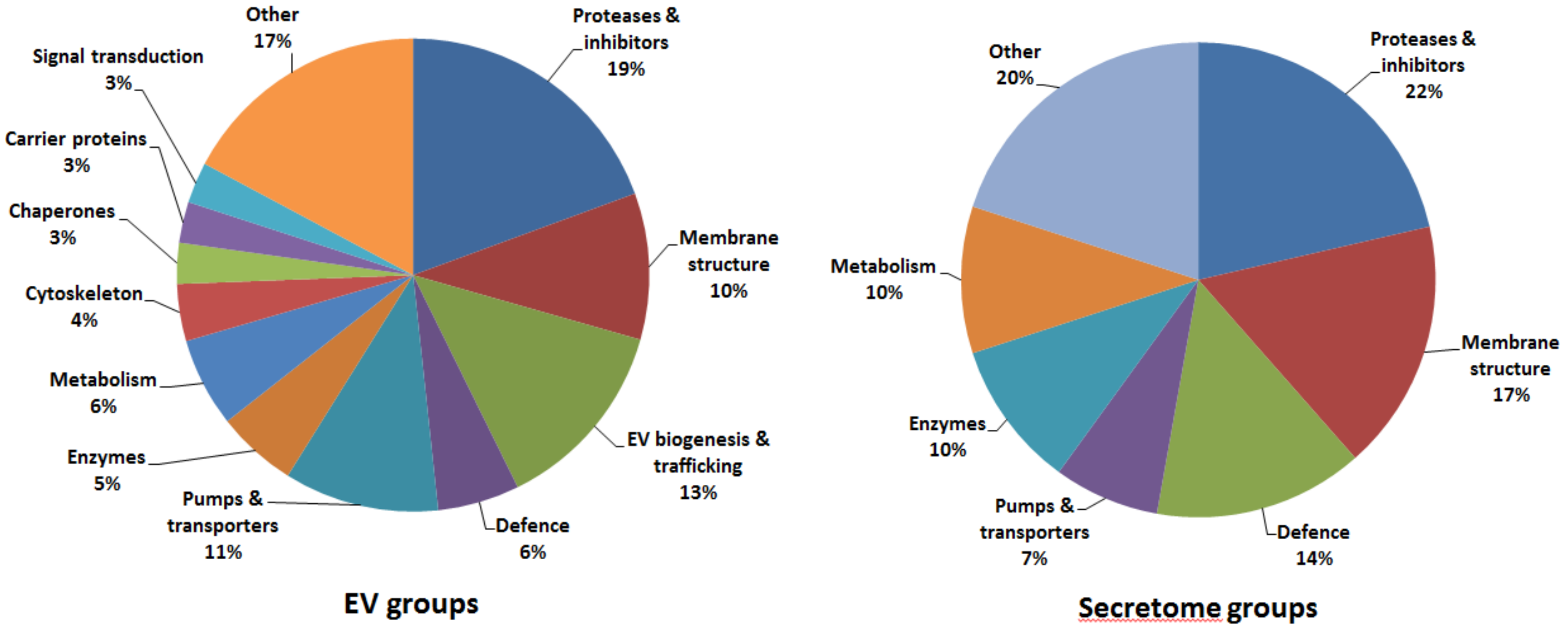

Supplement: Supplemental Data [file 10.1074_M115.053934_mcp.M115.053934-3.pdf]
