# Supplementary material for: The Extracellular Vesicles of the Helminth Pathogen, Fasciola hepatica: Biogenesis Pathways and Cargo Molecules Involved in Parasite Pathogenesis
Source: Mol Cell Proteomics. 2015 Oct 20;14(12):3258–73. doi: 10.1074/mcp.M115.053934 (PMC4762619; doi:10.1074/mcp.M115.053934)
Supplement: Supplemental Data [file 10.1074_M115.053934_mcp.M115.053934-4.docx]

**Supplementary Table 1:** Proteins identified from the total secretome of adult *F. hepatica* grouped according to function.

| **Protein** | **Identifier** | **Signal peptide^1^** | **Present in EVs** |
| --- | --- | --- | --- |
|  |  |  |  |
| ***Proteases & inhibitors*** |  |  |  |
| Lysosomal pro-Xaa carboxypeptidase | BN1106_s3518B000132 | Yes | ● |
| Cathepsin L1 | BN1106_s8490B000026 | Yes | ● |
| Lysosomal Pro-Xaa carboxypeptidase | BN1106_s1620B000120 | ND | ● |
| Cathepsin L1 | BN1106_s7289B000014 | Yes | ● |
| Cathepsin L2 | BN1106_s8098B000020 | Yes | ● |
| Cathepsin A | BN1106_s1241B000264 | No | ● |
| Cathepsin B4/5/7 | BN1106_s13444B000002 | ND | ● |
| Cathepsin L5 | BN1106_s3536B000078 | Yes |  |
| Legumain 3 | BN1106_s4223B000091 | Yes |  |
| Legumain 4/5 | BN1106_s1861B000097 | Yes | ● |
| Cathepsin B6/8 | BN1106_s793B000177 | Yes | ● |
| Dipeptidylpeptidase III | BN1106_s13034B000002 | No |  |
| Serpin B6 | BN1106_s122B000261 | No |  |
| Kunitz-type proteinase inhibitor | BN1106_s318B000274 | Yes | ● |
| Cystatin-1 | BN1106_s4651B000094 | No | ● |
|  |  |  |  |
| ***Defence*** |  |  |  |
| GST mu 26 | BN1106_s4479B000057 | No |  |
| GST mu 29 | BN1106_s3595B000059 | No |  |
| Saposin-like protein SAP-3 | BN1106_s10326B000017 | ND | ● |
| HDM1 | BN1106_s2101B000084 | Yes |  |
| GST sigma | BN1106_s1081B000242 | No | ● |
| Peroxiredoxin | BN1106_s1614B000280 | Yes | ● |
| Thioredoxin | BN1106_s4026B000080 | No | ● |
| GST mu 27 | BN1106_s7830B000018 | No |  |
| FABP Fh_2 | BN1106_s1228B000120 | No |  |
| Thioredoxin-glutathione reductase | BN1106_s9271B000022 | No |  |
|  |  |  |  |
| ***Membrane structure*** |  |  |  |
| Annexin | BN1106_s819B000364 | Yes | ● |
| Phospholipase A2 | BN1106_s4998B000033 | Yes |  |
| Annexin | BN1106_s945B000218 | No | ● |
| Heparan sulphate proteoglycan core protein | BN1106_s25B000189 | No |  |
| Fasciclin I-like protein | BN1106_s1995B000318 | No |  |
| Acid sphingomyelinase | BN1106_s1285B000159 | Yes | ● |
| Leucine-rich transmembrane protein | BN1106_s2848B000228 | Yes |  |
| Lysosome-associated membrane glycoprotein | BN1106_s1823B000148 | ND |  |
| Fasciclin I-like protein | BN1106_s1922B000120 | Yes |  |
| Otoferlin | BN1106_s4840B000058 | No | ● |
| CD63 antigen | BN1106_s4560B000072 | No | ● |
| Neurexin-1-alpha | BN1106_s462B000766 | No |  |
|  |  |  |  |
| ***Receptors & transporters*** |  |  |  |
| LDL receptor | BN1106_s60B000536 | ND |  |
| MDR p-glycoprotein 1 | BN1106_s2471B000098 | No | ● |
| LDL receptor | BN1106_s2354B000024 | ND |  |
| Niemann-Pick C2 protein | BN1106_s7353B000023 | Yes |  |
| ABCB1 | BN1106_s274B000296 | No | ● |
|  |  |  |  |
| ***Enzymes*** |  |  |  |
| Beta-galactosidase | BN1106_s5248B000014 | No |  |
| Acid phosphatase-like protein | BN1106_s1079B000448 | No |  |
| Ornithine aminotransferase | BN1106_s398B000241 | ND |  |
| Alpha-glucosidase | BN1106_s3173B000376 | No |  |
| Branched-chain amino acid aminotransferase | BN1106_s676B000138 | No |  |
| Aspartate aminotransferase | BN1106_s4453B000140 | No |  |
| Protein disulphide-isomerase | BN1106_s2763B000063 | Yes |  |
|  |  |  |  |
| ***Metabolism*** |  |  |  |
| Dihydrolipoamide dehydrogenase | BN1106_s8157B000032 | No |  |
| Glutamate dehydrogenase NAD(P)+ | BN1106_s8641B000016 | No | ● |
| Fructose-bisphosphate aldolase | BN1106_s4469B000065 | No | ● |
| Enolase | BN1106_s3227B000227 | No | ● |
| Triosephosphate isomerase | BN1106_s3213B000041 | No |  |
| Hexokinase A | BN1106_s175B000200 | No | ● |
|  |  |  |  |
| ***Other*** |  |  |  |
| Uncharacterised | BN1106_s8038B000016 | Yes | ● |
| Uncharacterised | BN1106_s1110B000106 | Yes | ● |
| Uncharacterised | BN1106_s3001B000131 | ND | ● |
| Uncharacterised | BN1106_s6821B000024 | ND | ● |
| Ferritin | BN1106_s709B000627 | No | ● |
| Ferritin | BN1106_s3950B000041 | No | ● |
| Collagen alpha-1(IV) chain | BN1106_s176B000279 | Yes |  |
| Filamin A | BN1106_s296B000186 | No |  |
| Ubiquitin-60S ribosomal protein L40 | BN1106_s6576B000103 | No | ● |
| Myoglobin 1 | BN1106_s284B000288 | No |  |
| DM9 domain-containing protein | BN1106_s5689B000026 | No | ● |
| 14-3-3 protein | BN1106_s3904B000042 | No | ● |
| EH domain-containing protein 1 | BN1106_s2100B000128 | No |  |
| Oestrogen-regulated protein EP45 | BN1106_s4565B000032 | No |  |

^1^Predicted using the SignalP algorithm; ND, not determined due to 5` truncation of transcript.
